# Hits-to-Lead Optimization of the Natural Compound 2,4,6-Trihydroxy-3-geranyl-acetophenone (tHGA) as a Potent LOX Inhibitor: Synthesis, Structure-Activity Relationship (SAR) Study, and Computational Assignment

**DOI:** 10.3390/molecules23102509

**Published:** 2018-09-30

**Authors:** Chean Hui Ng, Kamal Rullah, Faridah Abas, Kok Wai Lam, Intan Safinar Ismail, Fadzureena Jamaludin, Khozirah Shaari

**Affiliations:** 1 Laboratory of Natural Products, Institute of Bioscience, Universiti Putra Malaysia (UPM), Serdang 43400, Selangor, Malaysia; nch_chean@hotmail.com (C.H.N.); safinar@upm.edu.my (I.S.I.); 2 School of Pharmacy, Management and Science University (MSU), University Drive, Off Persiaran Olahraga, Seksyen 13, Shah Alam 40100, Selangor, Malaysia; 3 Faculty of Pharmacy, Universiti Kebangsaan Malaysia (UKM), Jalan Raja Muda Abdul Aziz, Kuala Lumpur 50300, Malaysia; kamalrullah@yahoo.co.id (K.R.); david_lam@ukm.edu.my (K.W.L.); 4 Nanotechnology and Catalysis Research Centre (NANOCAT), Institute of Postgraduates Studies, University of Malaya (UM), Kuala Lumpur 50603, Malaysia; 5 Department of Food Science, Faculty of Food Science and Technology, Universiti Putra Malaysia (UPM), Selangor Darul Ehsan, Serdang 43400, Selangor, Malaysia; faridah_abas@upm.edu.my; 6 Forest Research Institute (FRIM), Selangor Darul Ehsan, Kepong 52109, Malaysia; fadzureena@frim.gov.my

**Keywords:** Analogues, lipoxygenase, in-silico, ADMET, TOPKAT

## Abstract

A new series of 2,4,6-trihydroxy-3-geranyl-acetophenone (tHGA) analogues were synthesized and evaluated for their lipoxygenase (LOX) inhibitory activity. Prenylated analogues **4a**–**g** (half maximal inhibitory concentration (IC_50_) values ranging from 35 μM to 95 μM) did not exhibit better inhibitory activity than tHGA (**3a**) (IC_50_ value: 23.6 μM) due to the reduction in hydrophobic interaction when the alkyl chain length was reduced. One geranylated analogue, **3d**, with an IC_50_ value of 15.3 μM, exhibited better LOX inhibitory activity when compared to tHGA (**3a**), which was in agreement with our previous findings. Kinetics study showed that the most active analogue (**3e**) and tHGA (**3a**) acted as competitive inhibitors. The combination of in silico approaches of molecular docking and molecular dynamic simulation revealed that the lipophilic nature of these analogues further enhanced the LOX inhibitory activity. Based on absorption, distribution, metabolism, excretion, and toxicity (ADMET) and toxicity prediction by komputer assisted technology (TOPKAT) analyses, all geranylated analogues (**3a**–**g**) showed no hepatotoxicity effect and were biodegradable, which indicated that they could be potentially safe drugs for treating inflammation.

## 1. Introduction

Lipoxygenases (LOXs) are a family of nonheme iron-containing enzymes that are involved in the oxygenation of polyunsaturated fatty acids (PUFAs) containing *cis*–*cis* 1–4 pentadiene structures to give hydroperoxy derivatives. In general, LOXs can be found in plant and animal tissues [1]. They can be classified as 5-, 8-, 9-, 11-, 12-, and 15-LOXs based on their positional specificity of arachidonic acid oxidation [2].

LOXs are potential targets for rational drug design since they are involved in a variety of disorders, such as bronchial asthma, inflammation, cancer, and autoimmune diseases [3]. The human 5-LOX pathway is the source of potent proinflammatory mediators [4]. Leukotrienes (LTs), produced through the human 5-LOX pathway, are mediators for allergy and asthma, while cysteinyl leukotrienes (CysLTs), the metabolites of LTs, are known to sustain bronchoconstriction, hypersecretion of mucus, and airway edema, which can trigger asthma [5,6,7]. Hence, inhibitors for the human 5-LOX pathway are crucial in therapies for various inflammation-related diseases.

Synthesizing a molecule with specific human 5-LOX inhibition activity has emerged as a valuable therapeutic strategy. Thus, structural insight into the human 5-LOX active site and its interaction with ligands is essential in designing such compounds. However, due to the difficulties in obtaining the human enzyme in sufficiently purified form and the availability of soybean enzyme, most of the structural research had been done using soybean LOX as a biological screen [8,9].

**Figure 1:**
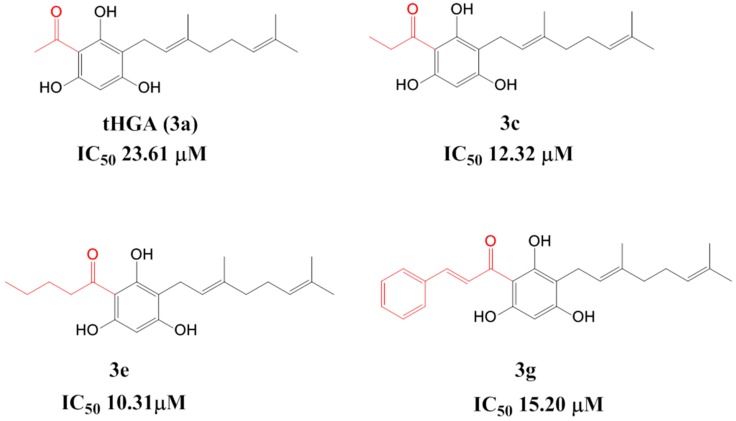
Half maximal inhibitory concentration (IC_50_) values of 2,4,6-trihydroxy-3-geranyl-acetophenone (tHGA) and analogues.

2,4,6-Trihydroxy-3-geranylacetophenone (tHGA) (**3a**) is a drug-like compound containing the phloroglucinol structural core as the bioactive principle (Figure 1). Our earlier bioassay-guided identification studies identified this compound in the medicinal plant *Melicope ptelefolia* [10]. This compound can also be synthesized via direct *C*-alkylation, using 2,4,6-trihydroxyacetophenone and geranyl bromide as the starting material [11]. Further exploration into the chemistry and pharmacology of tHGA revealed that it is a dual inhibitor of LOX and Cyclooxygenase (COX) [10,12]. When tested in an acute murine model, tHGA was as effective as Zileuton, a commercial LOX inhibitor, in preventing allergic airway inflammation [11].

Previously, we reported our synthesis and docking studies of geranylated analogues of tHGA [13]. In our preliminary SAR study, elongating the chain of acyl moieties and introducing an aromatic moiety significantly improved the inhibitory activity compared to tHGA. Biological evaluation of the analogues revealed that three targeted compounds, ((*E*)-1-(3-(3,7-dimethylocta-2,6-dienyl)-2,4,6-trihydroxyphenyl)propan-1-one (**3c**), (*E*)-1-(3-(3,7-dimethylocta-2,6-dienyl)-2,4,6-trihydroxyphenyl)pentan-1-one (**3e**), and (*E*)-1-(3-(*E*-3,7-dimethylocta-2,6-dienyl)-2,4,6-trihydroxyphenyl)-3-phenylprop-2-en-1-one (**3g**)), displayed better activity against soybean 15-LOX compared to tHGA (Figure 1). Preserving the lipophilic geranyl group improved the enzymatic activity compared to nongeranylated intermediates. However, the increased lipophilicity of the compounds reduced their solubility and led to a poor pharmacokinetic profile. This motivated us to synthesize a new series of tHGA analogues by shortening the geranyl group to a prenyl group, which we hypothesized could improve the solubility of the compounds.

## 2. Results and Discussion

### 2.1. Synthesis of 2,4,6-Trihydroxy-3-Geranylacetophenone (tHGA) Analogues

**Figure 2:**
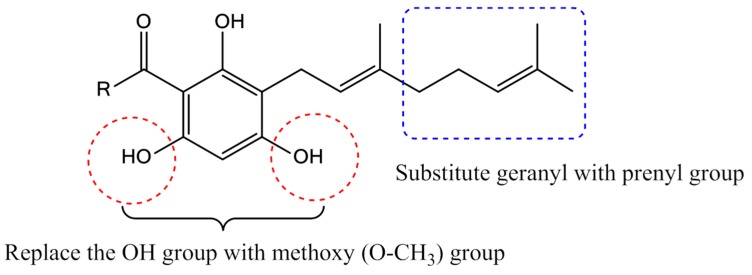
Modifications carried out on the geranyl and hydroxyl groups of tHGA analogues.

Previously, we reported the synthesis of the acylphloroglucinols (**2b**–**c**, **2e**–**g**) and geranylated analogues (**3a**–**c**, **3e**–**g**) and evaluated them for soybean-LOX inhibitory activity [13]. Our focus in this work is to reduce one isoprene unit from geranyl to prenyl and to replace the hydroxyl group in positions 4 and 6 of the phloroglucinol core by methoxy groups (Figure 2).

**Scheme 1:**
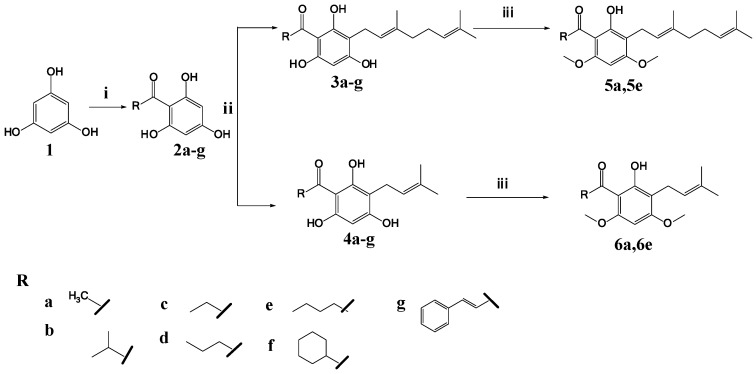
Reagents and conditions: (**i**) acyl chloride, anhydrous aluminum chloride, dichloromethane, hydrochloric acid, reflux 6 h; (**ii**) geranyl/prenyl bromide, anhydrous potassium carbonate, dry methanol, reflux 8 h; (**iii**) methyl iodide, anhydrous potassium carbonate, dimethylformamide, hydrochloric acid, reflux 9 h.

Prenylated analogues (**4a**–**g**) were synthesized by Friedel–Craft acylation and direct *C*-alkylation (Scheme 1). The acylphloroglucinols (**2b**–**g**) were first synthesized from phloroglucinol and acyl halides through Friedel–Craft acylation by using aluminum chloride as a catalyst [14]. Prenylated analogues were synthesized by reacting the acylphloroglucinols (**2a**–**g**) with prenyl bromide through direct *C*-alkylation, using potassium carbonate as a base in dry methanol. A geranylated analogue (**3d**) was also synthesized, by reacting acylphloroglucinol (**2d**) with geranyl bromide [11]. In order to investigate the importance of the hydroxyl group to the bioactivity, the hydroxyl group was methylated using methyl iodide as a methylating agent, as described by Smith (2005), to yield compounds **5a**, **5e** and **6a**, **6e** [15]. The structures of the seven prenylated, one geranylated, and four methylated analogues were established on the basis of ^1^H-NMR, ^13^C-NMR, and MS spectral data (Supplementary Materials).

### 2.2. Soybean LOX-1 Inhibition Assays

The prenylated analogues (**4a**–**g**) demonstrated moderate dose-dependent LOX inhibition, with IC_50_ values ranging from 35.1 to 95.4 μM. Geranylated analogue **3d** had an IC_50_ value of 15.3 μM. Unfortunately, the 15-LOX inhibitory activity of the methylated compounds (**5a**, **5e**, **6a**, and **6e**) could not be carried out due to insolubility of the compounds. To further understand the binding affinity of the analogues, nordihydroguaiaretic acid (NDGA), a known soybean LOX inhibitor, was used as reference standard (Table 1).

In comparison to geranylated compounds **3a**–**g** (IC_50_ = 10.3–26.3 μM), prenylated compounds **4a**–**g** were less active in their anti-inflammatory effect (IC_50_ = 35.1–95.4 μM). The reduction by one isoprene unit (geranyl to prenyl) reduced the lipophilicity of the compounds, which indirectly also reduced the inhibitory effect, as lipophilicity is an important physiochemical property for LOX inhibition [16,17,18,19]. Compound **3d** (IC_50_ = 15.3 μM), with the longer acyl chain, showed better inhibitory activity than tHGA (IC_50_ = 23.6 μM), which is in agreement with our previous findings [13].

The SAR pattern for the prenylated compounds was similar to that of the geranylated compounds, where the longest aliphatic chain length on the acyl substituent, as in compound **4e**, was the most active inhibitor (IC_50_ value 35.1 μM) among the compounds. Replacement of the aliphatic chain by a cyclohexyl ring and branched acyl substituent, as in compounds **4b** and **4f**, were also found to impart less potency than the synthetic analogues. However, compound **4g**, with an aromatic ring as the acyl substituent, was not as active as the geranylated series. This may be due to the insolubility of the compound when performing the assay. Among the active analogues of tHGA (**3a**), four compounds (**3c**–**e** and **3g**) exhibited higher inhibitory activity than the parent molecule, tHGA (**3a**). The most active compound, **3e** (IC_50_ = 10.3 μM), was 100-fold less active when compared to the standard drug, NDGA (IC_50_ = 0.1 μM).

The most active compound, **3e**, and the parent compound, tHGA (**3a**), were subjected to further in silico study of their probable mechanism of inhibition. Structural-based information for a protein alone is not enough to understand the binding affinity of the enzyme inhibitor. For a deeper understanding of how an inhibitor works with an enzyme, kinetic studies should be conducted in order to yield more useful information. In the present study, the type of inhibition was deduced from two plots, nonlinear regression Michaelis–Menten enzyme kinetics and the corresponding Lineweaver–Burk double reciprocal plots, based on the parameters *K_m_* (Michaelis–Menten constant) and *V_max_* (maximum enzyme velocity).

**Figure 3:**
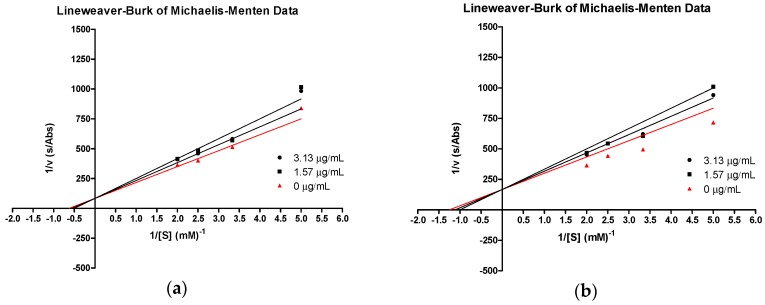
Lineweaver–Burk plot of 13-Hydroperoxyoctadeca-9,11-dienoic acid (13-HPOD) generated by soybean LOX-1 in the presence of (**a**) compound **3e** and (**b**) tHGA (**3a**) at 25 °C, pH 9.0. Concentrations of substrate used were 0.2, 0.3, 0.4, and 0.5 mM.

Our results show that both tHGA (**3a**) and compound **3e** inhibited catalytic activity of the soybean 15-LOX as a function of increasing concentration of the compound. The kinetic behavior of the enzyme followed Michaelis–Menten kinetics. The inhibition kinetics, analyzed by a Lineweaver–Burk plot, showed that tHGA and compound **3e** shared a competitive type of inhibition (Figure 3a,b), since increasing the inhibitor concentration resulted in a common intercept above the 1/*V_max_* axis with different slope. The *K_m_* for both compounds increased with increasing concentrations of inhibitor. However, the *V_max_* values remained constant.

Since compounds **3e** and tHGA (**3a**) are competitive inhibitors, there were no changes in maximal velocity. Hence, to calculate the inhibitor constant (*K_i_*), which is a measure of enzyme–inhibitor affinity or potency of different inhibitors, the slope of the Lineweaver–Burk plot was plotted against the inhibitor concentrations, where the intercept on the inhibitor axis gave the *K_i_* value [20]. The obtained *K_m_*, *V_max_*, and *K_i_* values are shown in Table 2. The inhibitor constant (*K_i_*) for compound **3e** (*K_i_* = 36.1) is lower than that of tHGA (**3a**) (*K_i_* = 41.1), which suggests that compound **3e** has higher affinity toward the enzyme than tHGA (**3a**).

### 2.3. Homology Modeling of Soybean LOX-1

In our previous study, docking studies were performed using the soybean LOX-3 crystal structure (PDB ID: 1IK3), although soybean LOX-1 was used in the actual experiment [13]. Since the LOX-1 crystal structure in the Protein Data Bank (PDB) library only exists in a close conformation, this negated our attempt to dock our compounds on the active site. To overcome this, a homology model approach was adopted by building a soybean LOX-1 model based on the template open-conformation structure of soybean LOX-3 (PDB ID: 1IK3).

Briefly, to initiate the model-building process, we used SWISS-MODEL, an automated protein homology-modelling server, to predict the open conformation of LOX-1. The pairwise alignment of the protein target and the template sequence is shown in Supplementary Figure S37. Since LOX-1 and LOX-3 share a high sequence similarity of 73.13%, this allowed us to accurately predict the position of the essential iron catalytic site (active site) in the model. The obtained model was then verified with PROCHECK analysis. Ramachandran plot of the minimized model revealed that 98.9% of the residues were located in the allowed regions (77.9% most favored), while 1.1% (eight residues) were outside the allowed regions (Supplementary Figure S38). Note that residues that were located outside the allowed regions were far from the substrate-binding domain, indicating that they may not have affected the ligand–protein binding simulations. Thus, it was concluded that the obtained final model of LOX-1 was satisfactory.

Although there were significant differences in terms of size, sequence, and substrate preference among the LOX isoenzymes, the overall fold and geometry of the essential nonheme iron-binding site were highly conserved [21,22]. In particular, the structural geometry of the iron-binding site for soybean LOX-1 comprises four amino acid ligands: the imidazole *N*-atoms of three histidine residues (His499, His504, and His690) and the carboxylate oxygen of the *C*-terminal Asn694, which chelated with the Fe^3+^. Based on the superimposition of soybean LOX-3 (PDB ID: 1IK3) over the soybean LOX-1 structure, the active site can be defined as a sphere of 10 Å centered on the inhibitor co-crystallized soybean LOX-3. Knowledge of the orientation of the essential amino acid residues in the active site will provide useful insights for the process of designing potent inhibitors.

### 2.4. Molecular Docking Analysis

Compounds **3e**, **4e**, and **3a** (tHGA) were docked to the homology model’s active site by cDOCKER. Compound **3e** exhibited the highest binding interaction energy of −50.28 kcal/mol compared to compound **4e**, with −42.65 kcal/mol, and tHGA (**3a**) with −43.03 kcal/mol. These binding interaction energies were well correlated with the bioassay results.

**Figure 4:**
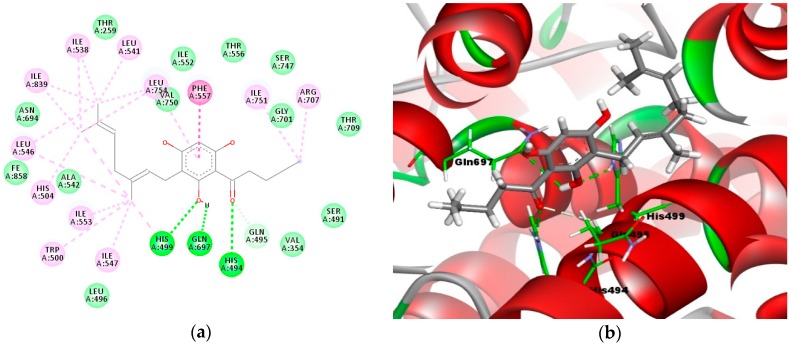
Molecular binding models of compound **3e** in the active site of the LOX-1 homology model: (**a**) 2D diagram; (**b**) 3D diagram. In (**a**), green line indicates hydrogen-bonding interactions, light green line indicates covalent bonding, pink line indicates hydrophobic interactions, and purple line indicates π–π interactions. In (**b**), the atoms are colored gray for carbon, red for oxygen, blue for nitrogen, and white for hydrogen; amino acid residues are in green.

The results (Figure 4a,b) show that compound **3e** formed three important hydrogen bonding interactions with amino acid residues His494, His499, and Gln697. In the 2D models (Figure 4a), the carbonyl moiety formed a 2.3 Å hydrogen bond with His494 (C–O----H–N), while the hydroxyl group of the phloroglucinol moiety formed two hydrogen bonds with His499 (H–O----H–N with a distance of 2.1 Å) and Gln697 (O–H----O–C with a distance of 2.5 Å). In addition, the carbonyl moiety also formed a 3.1 Å carbon–hydrogen bond with Gln495 (O–C----H–C). The alkyl groups of the geranyl and acyl side chains were also involved in a series of hydrophobic interactions with residues Ile538, Leu541, Leu754, Ile839, Leu546, His504, Ile553, Trp500, Ile547, His499, Ile751, and Arg707.

**Figure 5:**
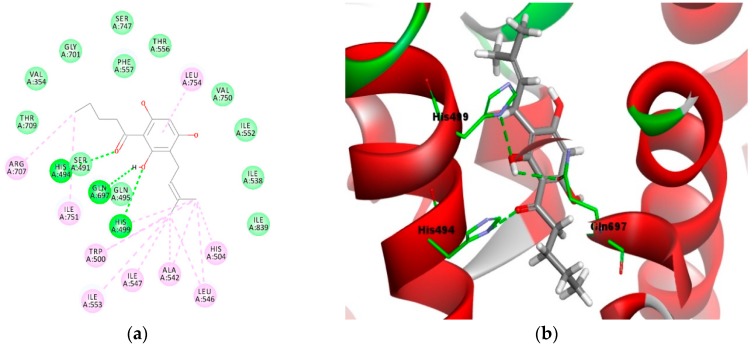
Molecular binding models of compound **4e** in the active site of the LOX-1 homology model: (**a**) 2D diagram; (**b**) 3D diagram. In (**a**), green line indicates hydrogen-bonding interactions, light green line indicates covalent bonding, pink line indicates hydrophobic interactions, and purple line indicates π–π interactions. In (**b**), atoms are colored gray for carbon, red for oxygen, blue for nitrogen, and white for hydrogen; amino acid residues are in green.

Similarly, the aromatic ring of the phloroglucinol moiety (Figure 5a,b) formed π–π and π–alkyl interactions with both Phe557 and Leu754. Figure 5a,b depict the binding of the most active prenylated compound **4e** in the active site of the LOX-1 homology model for comparison. As observed in the 2D model (Figure 5a), the oxygen atom of the carbonyl moiety formed a 3.2 Å hydrogen bond with His494 (C–O----H–N). The hydroxyl group of the phloroglucinol moiety formed two 2.7Å and 2.8 Å hydrogen bonds with the amino acid residues Gln697 (O–H----O–C) and His499 (H–O----N–C). As with compound **3e**, the alkyl groups from the acyl and prenyl side chains of compound **4e** were engaged in hydrophobic contact with residues Arg707, Ile751, His499, Trp500, Ile547, Ile553, Ala542, Leu546, and His504. In addition, a π–alkyl interaction between the aromatic ring of the phloroglucinol moiety with Leu754 was also observed.

**Figure 6:**
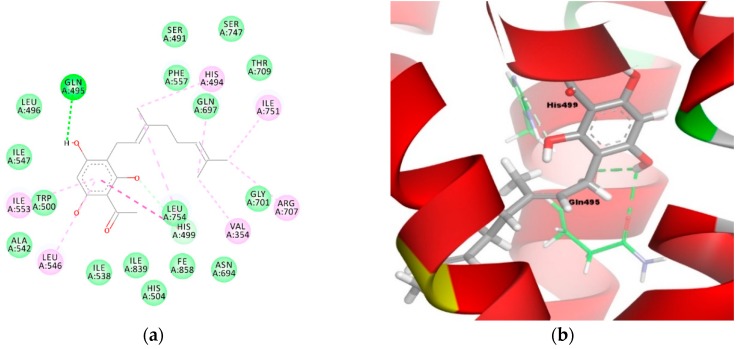
Molecular binding models of tHGA (**3a**) in the active site of the LOX-1 homology model: (**a**) 2D diagram; (**b**) 3D diagram. In (**a**), green line indicates hydrogen-bonding interactions, light green line indicates covalent bonding, pink line indicates hydrophobic interactions, and purple line indicates π–π interactions. In (**b**), atoms are colored gray for carbon, red for oxygen, blue for nitrogen, and white for hydrogen; amino acid residues are in green.

On the other hand, tHGA (**3a**) was stabilized by the hydrogen bonding interactions between the hydroxyl group of the phloroglucinol moiety with amino acid residue Gln495 (O–H----O–C with distances of 2.4 Å and 2.5 Å, respectively) and a 2.5 Å carbon-hydrogen bonding interaction with His499 (O–H----C–N) (Figure 6a,b). The geranyl moiety of tHGA (**3a**) formed a hydrophobic cluster with His494, His499, Ile751, Arg707, and Val354. In addition, the phloroglucinol moiety of tHGA (**3a**) formed another hydrophobic cluster with Ile553, Leu546, and His499 residues. Apart from these, the aromatic ring of tHGA (**3a**) also had a π–alkyl interaction with residues Ile553 and Leu546 and a π–π interaction with residue His499.

### 2.5. Molecular Dynamics Simulation

**Figure 7:**
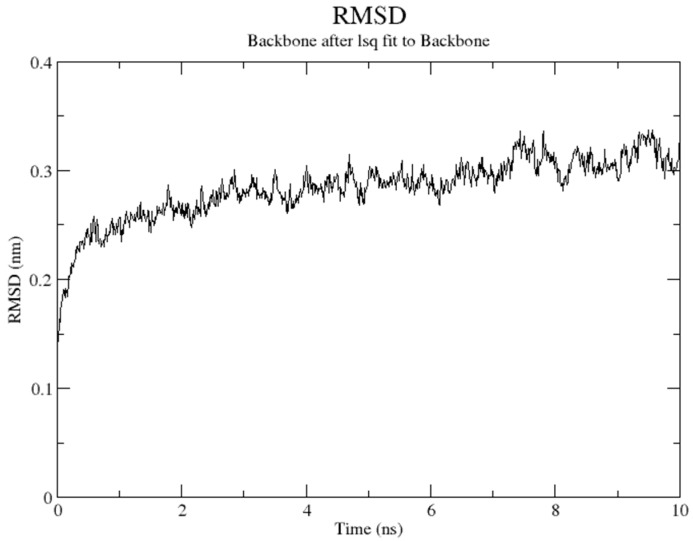
Total root-mean square deviation (RMSD) evolution along simulation time.

Molecular dynamics (MD) simulation plays an important role in drug discovery, as it allows drug designers to have clear insight into the movement of ligand in a protein binding site. In this study, MD simulations were started with the parameterization of ligands by using the Automated Topology Builder (ATB). The prepared complex was then pretreated, followed by restraints positioned on residues 499, 504, 690, and 694 (residues chelate with Fe atom). Figure 7 shows that the overall structure of the soybean LOX-1 model was well equilibrated after 1 ns, with a root-mean square deviation (RMSD) of 3.0 Å for the backbone atoms. It is clear that the position and orientation of compounds **3e**, **4e**, and tHGA (**3a**) in the binding site were slightly different than the initial input at the end of the simulation, as shown in Supplementary Figures S39–S41. The simulation results revealed that the inhibitors (**3e**, **4e**, and tHGA (**3a**)) may bind in a similar manner to the LOX-1 binding site. Notably, the alkyl side chains of the compounds were engaged in a series of hydrophobic contacts with five amino acid residues, His499, Trp500, Ala542, Ile547, and Leu754.

**Figure 8:**
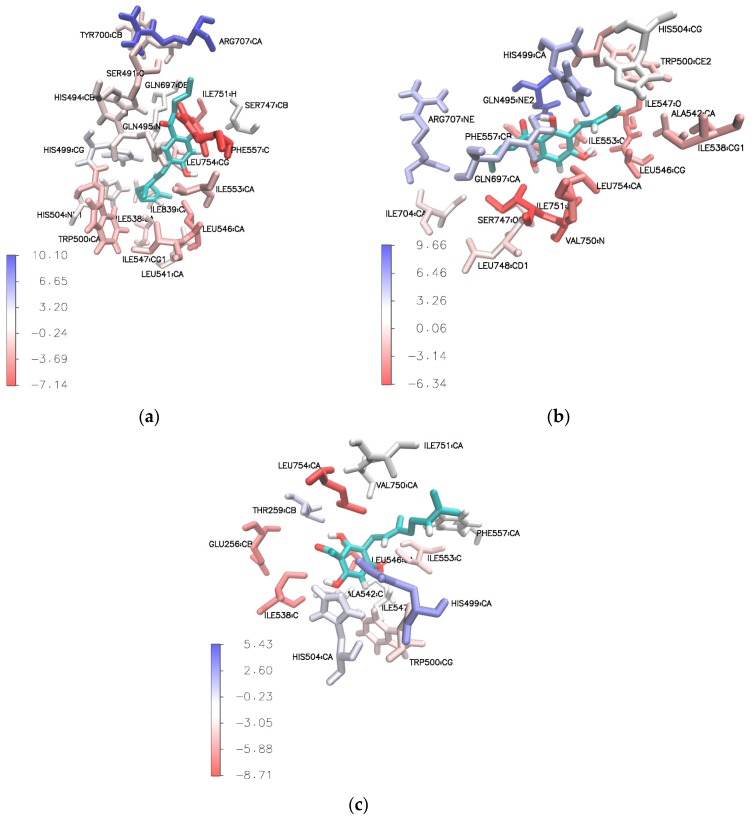
Stereo view of binding sites of (**a**) compound **3e**, (**b**) compound **4e**, and (**c**) compound **3a** (tHGA) after simulation. Residues that favor binding interactions are colored in red; residues that are not favorable for binding interactions are shown in blue.

The Molecular Mechanics Poisson-Boltzmann Surface Area (MM-PBSA) calculation was further employed to evaluate the free energy decomposition at the atomic level, especially the Van der Waals and electrostatic energy contributions. This calculation is extremely useful in understanding ligand binding behavior in binding sites. Residues that favor binding interactions are colored in red, while residues that are not favorable for binding interactions are shown in blue (Figure 8).

The results show that the phloroglucinol aromatic ring of the most active compound (**3e**) formed a stacked π–π interaction with Phe557 taken from the final ns of the simulation, as shown in Supplementary Figures S42 and S43. This particular amino acid residue could play an important role in the placement of the compound in the active site via hydrophobic interaction (π–π interaction) with significant total free binding interaction energy (∆G_bind_ = −7.14 kcal/mol) (Supplementary Table S1).

Based on the calculated residue-based energy decomposition, two amino acid residues, Ile751 (∆G_bind_ = −5.13 kcal/mol) and Leu546 (∆G_bind_ = −4.04 kcal/mol), were identified to have favorable interaction with the ligand via π–alkyl interaction (Supplementary Table S1).

Similar results were also observed for compound **4e**. As shown in Supplementary Table S2, the amino acid residues that contributed to significant binding interaction energy with compound **4e** were Ile751 (∆G_bind_ = −6.34 kcal/mol), Val750 (∆G_bind_ = −6.13 kcal/mol), Ile547 (∆G_bind_ = −5.18 kcal/mol), and Leu546 (∆G_bind_ = −4.51 kcal/mol). The acyl side chain formed a π–alkyl interaction with Ile751, while the prenyl side chain formed π–alkyl interactions with Ile547 and Ile546 (refer to Supplementary Figures S44 and S45). On the other hand, the aromatic ring was engaged in π–alkyl interactions with Val750 and Ile751, and formed a π–sigma interaction with Val750 (Supplementary Figures S44 and S45). Note that residue Val750, which played a major role in hydrophobic interactions, was not found in the docking results. The abovementioned amino acid residues are shown in red in Figure 8b.

From our observation, tHGA (**3a**) was stabilized by three important amino acid residues, Leu754, Leu546, and Ile538, which contributed to significantly high values of total free binding interaction energy, with values of −8.71 kcal/mol, −7.45 kcal/mol, and −6.63 kcal/mol, respectively (Supplementary Table S3). These amino acid residues were involved in the hydrophobic interaction with the hydrophobic geranyl side chain and phloroglucinol aromatic ring of tHGA (**3a**) (refer to Supplementary Figures S46 and S47). In addition, residue Glu256 also contributed significant total free binding interaction energy (∆G_bind_ = −6.90 kcal/mol) from the carbon–hydrogen bonding interaction with the carbonyl moiety of tHGA (**3a**) (refer to Supplementary Figures S46 and S47).

Table 3 summarizes the binding free energy (∆G_bind_) for compounds **3e**, **4e**, and tHGA (**3a**). The most lipophilic geranylated compound **3e** (−150.71 kJ/mol), with larger hydrophobic interactions with the adjacent amino acid residues, had the highest binding free interaction energy when compared to the less lipophilic prenylated compound **4e** (−111.79 kJ/mol) and tHGA (**3a**) (−148.04 kJ/mol). The major favorable contributors to inhibitor binding were Van der Waals energy (∆E_vdw_) and nonpolar solvation energy (∆G_np_). Although electrostatic energy (∆E_ele_) was as high as nonpolar solvation energy (∆G_np_), the unfavorable polar solvation energy (∆G_pb_) minimized its effect. The results suggest that the Van der Waals interaction was the main contributor to the binding affinity of these compounds (**3e**, **4e**, and tHGA (**3a**)). Overall, compound **3e** had larger hydrophobic interaction contact when compared to compound **4e** and tHGA (**3a**). This observation correlates well with the bioassay result, in which compound **3e** is a more potent inhibitor (IC_50_ = 10.31 μM, *K_i_* = 36.08) than tHGA (**3a**) (IC_50_ = 23.61 μM, *K_i_* = 41.06) and compound **4e** (IC_50_ = 35.10 μM).

Nevertheless, the crucial iron-chelating His499 residue was shown to interact with compounds **3e**, **4e**, and tHGA (**3a**) during the simulation. This further led to distortion of the iron active site geometry. In fact, the kinetics results suggest that both tHGA (**3a**) and the most active compound **3e** compete with the substrates without chelating with the iron metal.

### 2.6. ADMET Analysis

Absorption, distribution, metabolism, excretion, and toxicity (ADMET) prediction is an important tool for compound selection and prioritization in rational drug design. The information helps reduce the time and computational cost of screening compound libraries to select compounds feasible for synthesis and further testing [23,24]. The results are presented in Table 4.

Based on the ADMET findings, tHGA (**3a**) and all prenylated compounds (**4a**, **4b**, **4c**, **4d**, and **4e**), except for compounds **4f** and **4g,** showed good intestinal absorption and good aqueous solubility. In addition, tHGA (**3a**) and compounds **4b**–**g** could be potent candidates for the treatment of central nervous system inflammatory disorders due to their moderate to high blood–brain barrier (BBB) penetration. Compound **4a** showed low BBB penetration.

For protein plasma binding (PBB), all compounds were expected to be highly bound to protein plasma, which implied that dosing issues must be addressed in order to achieve desirable therapeutic effects. In addition, compounds **3f**, **3g**, **4a**–**c**, and **4f**–**g** were noninhibitors for CYP2D6, indicating their tolerance toward CYP2D6. On a positive note, none of the geranylated compounds (tHGA (**3a**), **3b**–**g**) showed hepatotoxicity effect.

### 2.7. TOPKAT Analysis

The major goal in medicinal chemistry is to develop safe drugs with few side effects. Thus, toxicology studies, especially during the preclinical and clinical stages, must be performed in order to assess the potential toxic effects of drugs. Toxicity prediction by komputer assisted technology (TOPKAT) is a common tool for predicting the potential ecotoxicity, toxicity, mutagenicity, and reproductive or developmental toxicity of drug candidates.

The 14 selected compounds from ADMET analysis were further screened with TOPKAT for the following toxicity prediction properties: aerobic biodegradability, Ames mutagenicity, rodent carcinogenicity, ocular irritancy, skin irritancy, and skin sensitization. The results are presented in Table 5. It was found that, in general, all compounds were skin sensitizers but nonmutagenic, noncarcinogenic, and nonirritant. All of the compounds were predicted to be biodegradable, except for compound **4g**. Nine of the compounds (**3a**–**g**, **4a** and **4g**) were predicted to be ocular nonirritants.

## 3. Materials and Methods

### 3.1. Synthesis of 2,4,6-Trihydroxy-3-Geranylacetophenone (tHGA) Analogues

#### 3.1.1. General Methods

All reagents and solvents used were analytical grade. Column chromatography was performed with Merck 7734 (0.040–0.063 mm) or Merck 9385 (0.063–0.200 mm) silica gel, while elutes were analyzed with Merck TLC silica gel 60 F_254_. Nuclear magnetic resonance (NMR) was recorded in CD_3_OD or CDCl_3_ using a 500 MHz Varian spectrometer, and mass analysis was done with a Shimadzu QP5050A mass spectrometer. Melting points were determined using a Fisher–Johns hot stage melting point apparatus equipped with microscope.

#### 3.1.2. Two-Step Reaction: Friedel–Crafts Acylation and Direct *C*-Alkylation (**3d**, **4a**–**g**)

Friedel–Crafts acylation: Small amounts of phloroglucinol (0.05 mol) and anhydrous aluminum chloride (0.1 mol) were first dissolved in dichloromethane (50 mL) and kept heated for 1 h at 40 °C. Acyl chloride (0.05 mole) was added dropwise into the reaction mixture, refluxed for 8 h, and quenched with 0.05 mole of HCl upon completion of the reaction. After the reaction was completed, the reaction mixture was extracted with ethyl acetate and concentrated under reduced pressure. The residues were subjected to column chromatography using silica gel eluted with hexane/ethyl acetate (10:1) [25]. Direct *C*-alkylation: A well-mixed mixture of acylphloroglucinol (1 mmol), geranyl bromide or prenyl bromide (1 mmol), and anhydrous potassium carbonate (0.5 mmol) in dry methanol (3 mL) was refluxed for 8 h. After completion of the reaction, excess methanol was removed through high vacuum and the residues were extracted with ethyl acetate. The organic layers were combined, dried, and concentrated. Column chromatography over silica gel eluted with petroleum ether/ethyl acetate (10:1) gave geranylated/prenylated compounds [11].

*(E)-1-(3-(3,7-dimethylocta-2,6-dienyl)-2,4,6-trihydroxyphenyl)butan-1-one* (**3d**). Light yellow solid (9.7%), m.p. 116–118 °C. ^1^H-NMR (500 MHz, Methanol-d_4_) d 5.89 (1H, s, H-4), 5.17 (1H, t, *J* = 6.5 Hz, H-2″), 5.05 (1H, t, *J* = 6.6 Hz, H-7″), 3.18 (2H, d, *J* = 7.0 Hz, H-1″), 3.01 (2H, t, *J* = 7.2 Hz, H-2′), 2.04 (2H, q, *J* = 7.4 Hz, H-6″), 1.93 (2H, t, *J* = 7.4 Hz, H-5″), 1.73 (3H, s, H-4″), 1.66–1.70 (2H, m, H-3′), 1.60 (3H, s, H-10″), 1.55 (3H, s, H-9″), 0.97 (3H, t, *J* = 7.4 Hz, H-4′). ^13^C-NMR (126 MHz, methanol-d_4_) 205.96 (C-1′), 163.55 (C-3) 162.14 (C-1), 160.01 (C-5), 133.27 (C-3″), 130.54 (C-8″), 124.09 (C-7″), 123.22 (C-2″), 106.63 (C-2), 103.85 (C-6), 93.43 (C-4), 45.46 (C-2′), 39.49 (C-5″), 26.30 (C-6″), 24.41 (C-10″), 20.65 (C-1″), 18.25 (C-9″), 16.27 (C-3′), 14.77 (C-4″), 13.0 (C-4′). DIP: *m*/*z* 332.15.

*1-(2,4,6-trihydroxy-3-(3-methylbut-2-enyl)phenyl)ethanone* (**4a**). Light yellow solid (12.5%), m.p. 164–166 °C. ^1^H-NMR (500 MHz, methanol-d_4_) d 5.89 (1H, s, H-4), 5.16 (1H, t, *J* = 7.1 Hz, H-2″), 3.16 (2H, d, *J* = 7.1 Hz, H-1″), 2.59 (3H, s, H-2′), 1.73 (3H, s, H-5″), 1.63 (3H, s, H-4″). ^13^C-NMR (126 MHz, Methanol-d_4_) d 203.14 (C-1′), 163.36 (C-3), 162.47 (C-1), 160.36 (C-5), 129.63 (C-3″), 123.09 (C-2″), 106.47 (C-2), 104.07 (C-6), 93.32 (C-4), 31.39 (C-2′), 24.51 (C-4″), 20.68 (C-1″), 16.40 (C-5″). DIP: *m*/*z* 236.10.

*2-methyl-1-(2,4,6-trihydroxy-3-(3-methylbut-2 enyl)phenyl)propan-1-one* (**4b**). Yellow solid (10.8%), m.p. 142–144 °C. ^1^H-NMR (500 MHz, methanol-d_4_) d 5.88 (1H, s, H-4), 5.16 (1H, t, *J* = 6.5 Hz, H-2″), 3.95–4.03 (1H, m, H-2′), 3.17 (2H, d, *J* = 7.1 Hz, H-1″), 1.73 (3H, s, H-4″), 1.63 (3H, s, H-5″), 1.12 (6H, d, *J* = 6.9 Hz, H-3′, 4′). ^13^C-NMR (126 MHz, Methanol-d_4_) 210.30 (C-1′), 163.85 (C-3), 161.98 (C-5), 159.58 (C-1), 129.60 (C-3″), 123.16 (C-2″), 106.71 (C-6), 103.07 (C-2), 93.53 (C-4), 38.48 (C-2′), 24.52 (C-5″), 20.78 (C-1″), 18.36 (C-3′, 4′), 16.41 (C-4″). DIP: *m*/*z* 264.10.

*1-(2,4,6-trihydroxy-3-(3-methylbut-2-enyl)phenyl)propan-1-one* (**4c**). Light brown solid (8.0%), m.p. 138–140 °C. ^1^H-NMR (500 MHz, methanol-d_4_) d 5.88 (1H, s, H-4), 5.16 (1H, t, *J* = 7.5 Hz, H-2″), 3.17 (2H, d, *J* = 7.1 Hz, H-1″), 3.05 (1H, q, *J* = 7.3 Hz, H-2′), 1.73 (3H, s, H-4″), 1.63 (3H, s, H-5″), 1.12 (3H, t, *J* = 7.3 Hz, H-3′). ^13^C-NMR (126 MHz, methanol-d_4_) 206.52 (C-1′), 163.40 (C-3), 162.04 (C-5), 160.03 (C-1), 129.61 (C-3″), 123.15 (C-2″), 106.55 (C-6), 103.66 (C-2), 93.38 (C-4), 36.58 (C-2′), 24.52 (C-5″), 20.73 (C-1″), 16.41 (C-4″), 7.94 (C-3′). DIP: *m*/*z* 250.10.

*1-(2,4,6-trihydroxy-3-(3-methylbut-2-enyl)phenyl)butan-1-one* (**4d**). Light yellow solid (9.1%), m.p. 138–140 °C. ^1^H-NMR (500 MHz, methanol-d_4_) d 5.88 (1H, s, H-4), 5.16 (1H, t, *J* = 7.1 Hz, H-2″), 3.16 (2H, d, *J* = 7.1 Hz, H-1″), 2.99–3.02 (2H, m, H-2′), 1.73 (3H, s, H-4″), 1.68 (2H, dd, *J* = 7.4, 14.8 Hz, H-3′), 1.63 (3H, s, H-5″), 0.97 (3H, t, *J* = 7.4 Hz, H-4′). ^13^C-NMR (126 MHz, methanol-d_4_) 205.92 (C-1′), 163.53 (C-3), 162.11 (C-1), 160.00 (C-5), 129.60 (C-3″), 123.15 (C-2″), 106.57 (C-2), 103.82 (C-6), 93.40 (C-4), 45.45 (C-2′), 24.51 (C-5″), 20.72 (C-1″), 18.21 (C-4″), 16.40 (C-3′), 12.98 (C-4′). DIP: *m*/*z* 264.10.

*1-(2,4,6-trihydroxy-3-(3-methylbut-2-enyl)phenyl)pentan-1-one* (**4e**). Light yellow solid (5.5%), m.p. 133–135 °C. ^1^H-NMR (500 MHz, methanol-d_4_) d 5.88 (1H, s, H-4), 5.16 (1H, t, *J* = 6.5 Hz, H-2″), 3.16 (2H, d, *J* = 7.1 Hz, H-1″), 3.01–3.04 (2H, m, H-2′), 1.73 (3H, s, H-5″), 1.60–1.63 (5H, m, H-3′, 4″), 1.35–1.41 (2H, m, H-4′), 0.94 (3H, t, *J* = 7.4 Hz, H-5′). ^13^C-NMR (126 MHz, methanol-d_4_) 206.14 (C-1′), 163.54 (C-3), 162.13 (C-1), 159.98 (C-5), 129.61 (C-3″), 123.14 (C-2″), 106.57 (C-2), 103.79 (C-6), 93.40 (C-4), 43.21 (C-2′), 27.16 (C-3′), 24.51 (C-5″), 22.31 (C-4′), 20.73 (C-1″), 16.41 (C-4″), 12.88 (C-5′). DIP: *m*/*z* 278.10.

*Cyclohexyl(2,4,6-trihydroxy-3-(3-methylbut-2-enyl)phenyl)methanone* (**4f**). Dark brown oil (6.5%). ^1^H-NMR (500 MHz, methanol-d_4_) d 5.87 (1H, s, H-4), 5.16 (1H, t, *J* = 7.2 Hz, H-2″), 3.70–3.74 (1H, m, H-2′), 3.16 (2H, d, *J* = 7.1 Hz, H-1″), 1.73–1.90 (4H, m, H-3′, 7′), 1.73 (3H, s, H-5″), 1.65 (3H, s, H-4″), 1.25–1.40 (6H, m, H-4′, 5′, 6′). ^13^C-NMR (126 MHz, methanol-d_4_) 209.39 (C-1′), 167.98 (C-3), 161.90 (C-5), 159.62 (C-1), 129.61 (C-3″), 123.16 (C-2″), 106.71 (C-2), 103.23 (C-6), 93.56 (C-4), 49.19 (C-2′), 29.39 (C-3′, 7′), 25.93 (C-4′, 5′, 6′), 24.51 (C-4″), 20.77 (C-1″), 16.41 (C-5″). DIP: *m*/*z* 304.10.

*(E)-3-phenyl-1-(2,4,6-trihydroxy-3-(3-methylbut-2-enyl)phenyl)prop-2-en-1-one* (**4g**). Yellow solid (13.9%), m.p. 203–205 °C. ^1^H-NMR (500 MHz, methanol-d_4_) d 7.49 (2H, d, *J* = 7.5 Hz, H-5′, 9′), 7.41 (2H, t, *J* = 7.5 Hz, H-6′, 8′), 7.36 (1H, d, *J* = 7.4 Hz, H-7′), 5.97 (1H, s, H-4), 5.43 (1H, d, *J* = 17.5 Hz, H-2′), 5.19 (1H, t, *J* = 7.1 Hz, H-2″), 3.20 (2H, d, *J* = 7.1 Hz, H-1″), 2.76 (1H, d, *J* = 17.5 Hz, H-3′), 1.75 (3H, s, H-5″), 1.65 (3H, s, H-4″). ^13^C-NMR (126 MHz, methanol-d_4_) 197.43 (C-1′), 166.02 (C-3), 162.50 (C-1), 162.38 (C-5), 140.63 (C-4′), 131.66 (C-3″), 129.72 (C-6′, 8′), 129.61 (C-7′), 127.36 (C-5′, 9′), 123.88 (C-2″), 109.83 (C-2), 103.25 (C-6), 95.48 (C-4), 80.46 (C-2′), 44.39 (C-3′), 25.99 (C-5″), 21.91 (C-1″), 17.88 (C-5″). DIP: *m*/*z* 323.95.

#### 3.1.3. Three-Step Reaction: Friedel–Crafts Acylation, Direct *C*-Alkylation, and Methylation (**5a**, **5e**, **6a**, and **6e**)

Methylation: A well-mixed mixture of geranylated/prenylated acylphloroglucinols (0.5 mmol) and K_2_CO_3_ (3 mmol), and 20 mL of dimethylformamide (DMF) were allowed to react, with constant stirring, for 20 min. Methyl iodide (2.3 mmol) was added dropwise into the reaction mixture and the mixture was heated for 9 h at 80 °C. Concentrated HCl (15 mL, 1 M) was added to quench the reaction, and it was stirred for another 10 min. The resultant products were extracted with dichloromethane (DCM) followed by cold water 3 times. The residue was purified using silica gel column chromatography (hexane:ethyl acetate = 10:1) [26].

*(E)-1-(3-(3,7-dimethylocta-2,6-dienyl)-2-hydroxy-4,6-dimethoxyphenyl)ethanone* (**5a**). Light yellow solid (35.0%), m.p. 72–74 °C. ^1^H-NMR (500 MHz, methanol-d_4_) d 6.16 (1H, s, H-4), 5.11 (1H, t, *J* = 7.7 Hz, H-2″), 5.03 (1H, t, *J* = 7.9 Hz, H-7″), 3.93 (3H, s, 2′′′), 3.90 (3H, s, H-1′′′), 3.21 (2H, d, *J* = 7.1 Hz, H-1″), 2.58 (3H, s, H-2′), 2.02 (2H, q, *J* = 7.9 Hz, H-6″), 1.92 (2H, t, *J* = 7.9 Hz, H-5″), 1.73 (3H, s, H-4″), 1.59 (3H, s, H-10″), 1.54 (3H, s, H-9″). ^13^C-NMR (126 MHz, methanol-d_4_) 203.39 (C-1′), 163.67 (C-3), 162.82 (C-1), 162.11 (C-5), 133.67 (C-3″), 130.52 (C-8″), 124.01 (C-7″), 122.69 (C-2″), 108.98 (C-2), 105.31 (C-6), 86.08 (C-4), 54.71 (C-1′′′), 54.66 (C-2′′′), 39.43 (C-5″), 31.86 (C-2′), 26.27 (C-6″), 24.39 (C-10″), 20.57 (C-1″), 16.25 (C-9″), 14.71 (C-4″). DIP: *m*/*z* 332.20.

*(E)-1-(3-(3,7-dimethylocta-2,6-dienyl)-2-hydroxy-4,6-dimethoxyphenyl)pentan-1-one* (**5e**). Brown oil (46.6%). ^1^H-NMR (500 MHz, methanol-d_4_) d 6.16 (1H, s, H-4), 5.10–5.13 (1H, m, H-2″), 5.01–5.04 (1H, m, H-7″), 3.93 (3H, s, 2′′′), 3.90 (3H, s, H-1′′′), 3.21 (2H, d, *J* = 7.0 Hz, H-1″), 2.97–3.00 (2H, m, H-2′), 2.03 (2H, dd, *J* = 6.6, 14.0 Hz, H-6″), 1.92 (2H, t, *J* = 7.5 Hz, H-5″), 1.73 (3H, s, H-4″), 1.63 (2H, dd, *J* = 7.5, 14.9 Hz, H-3′), 1.59 (3H, s, H-10″), 1.54 (3H, s, H-9″), 1.39 (2H, td, *J* = 7.2, 14.6 Hz, H-3′), 0.95 (3H, t, *J* = 7.4 Hz, H-5′). ^13^C-NMR (126 MHz, methanol-d_4_) 207.68 (C-1′), 164.77 (C-3), 164.37 (C-1), 163.18 (C-5), 135.05 (C-3″), 131.96 (C-8″), 125.45 (C-7″), 124.17 (C-2″), 110.48 (C-2), 106.48 (C-6), 87.49 (C-4), 56.13 (C-1′′′), 56.09 (C-2′′′), 45.23 (C-2′), 40.89 (C-5″), 28.40 (C-3′), 27.70 (C-6″), 25.88 (C-10″), 23.73 (C-4′), 22.08 (C-1″), 17.73 (C-9″), 16.19 (C-4″), 14.41 (C-5′). DIP: *m*/*z* 374.10.

*1-(2-hydroxy-4,6-dimethoxy-3-(3-methylbut-2-enyl)phenyl)ethanone* (**6a**). Light yellow solid (9.1%), m.p. 97–99 °C. ^1^H-NMR (500 MHz, methanol-d_4_) d 5.95 (1H, s, H-4), 5.17 (1H, t, *J* = 6.3 Hz, H-2″), 3.88 (3H, s, 2′′′), 3.88 (3H, s, H-1′′′), 3.26 (2H, d, *J* = 6.8 Hz, H-1″), 2.60 (3H, s, H-2′), 1.76 (3H, s, H-5″), 1.66 (3H, s, H-4″). ^13^C-NMR (126 MHz, methanol-d_4_) 203.26 (C-1′), 163.52 (C-3), 163.20 (C-1), 161.68 (C-5), 131.31 (C-3″), 122.66 (C-2″), 109.76 (C-2), 105.99 (C-6), 85.84 (C-4), 55.45 (C-1′′′), 55.33 (C-2′′′), 33.08 (C-2′), 25.76 (C-4″), 21.27 (C-1″), 17.71 (C-5″). DIP: *m*/*z* 264.10.

*1-(2-hydroxy-4,6-dimethoxy-3-(3-methylbut-2-enyl)phenyl)pentan-1-one* (**6e**). Light yellow solid (38.0%), m.p. 84–86 °C. ^1^H-NMR (500 MHz, methanol-d_4_) d 5.95 (1H, s, H-4), 5.18 (1H, t, *J* = 7.0 Hz, H-2″), 3.89 (3H, s, 2′′′), 3.88 (3H, s, H-1′′′), 3.26 (2H, d, *J* = 7.0 Hz, H-1″), 2.96–2.99 (2H, m, H-2′), 1.76 (3H, s, H-5″), 1.60–1.66 (5H, m, H-3′, 4″), 1.35–1.42 (2H, m, H-4′), 0.94 (3H, t, *J* = 7.3 Hz, H-5′). ^13^C-NMR (126 MHz, methanol-d_4_) 206.31 (C-1′), 163.62 (C-3), 162.88 (C-1), 161.42 (C-5), 131.39 (C-3″), 122.69 (C-2″), 109.96 (C-2), 106.09 (C-6), 85.82 (C-4), 55.45 (C-1′′′), 55.34 (C-2′′′), 44.20 (C-2′), 29.70 (C-3′), 27.07 (C-5″), 25.83 (C-4′), 22.66 (C-1″), 21.32 (C-4″), 14.04 (C-5′). DIP: *m*/*z* 306.05.

### 3.2. In Vitro Soybean 15-Lipoxygenase (LOX) Inhibition Assay

Briefly, sodium phosphate buffer (160 μL, 100 mM, pH 8.0), test sample (10 μL), and soybean 15-LOX (1.13.11.12) type I-B solution (20 μL, 320 U/well) were mixed and incubated at room temperature for 15 min. The substrate linoleic acid (10 μL, 0.6 mM) solution was added to initiate the reaction. End product from the enzymatic conversion was measured at absorbance of 234 nm over a period of 6 min and the assay was performed in triplicate. Dimethyl sulfoxide (DMSO) was used as a solvent to dissolve test samples and reference standard. Test concentrations in the range of 1.57 to 100 μg/mL were used to determine the IC_50_ values of the pure compounds, while test concentrations in the range of 0.00625 to 25 μg/mL were used to determine the IC_50_ value of the reference compound, nordihydroguaiaretic acid (NDGA). IC_50_ values were calculated by the nonlinear regression fitting curve of GraphPad Prism 5. The percentage of inhibition can be calculated by the following formula [10,11]:

$$\mathrm{Inhibitory}\%\;=\;\frac{\mathrm{ODcontrol}-\mathrm{ODsample}}{\mathrm{ODcontrol}}$$

Kinetics study: Enzyme kinetics was performed similar to the above inhibition assay with slight modification. In brief, sodium phosphate buffer (160 μL, 100 mM, pH 9.0), test sample (10 μL), and soybean 15-LOX (1.13.11.12) type I-B solution (20 μL, 320 U/well) were mixed and incubated for 15 min at room temperature. About 10 μL each of 4 linoleic acid substrate concentrations (0.2, 0.3, 0.4, and 0.5 mM) was added to initiate the enzymatic conversion. End product of the reaction was measured at 234 nm over a period of 10 min, with readings taken every 30 s. The type of inhibition (competitive, uncompetitive, noncompetitive) was determined by nonlinear regression Michaelis–Menten enzyme kinetics and the corresponding Lineweaver–Burk plots using 3 concentrations of each inhibitor: 0, 1.57, and 3.13 μg/mL. All experiments were performed in triplicate and the average values and standard errors were plotted. The *K_m_* and *V_max_* values were calculated from the nonlinear regression fitting curve of GraphPad Prism 5.

### 3.3. Homology Modeling

The target amino acid sequence of soybean LOX-1 (Accession ID: P08170) was taken from UniProtKB in FASTA format. Lipoxygenase-3 (soybean), sequestered from Research Collaboratory for Structural Bioinformatics (RCSB) PDB (PDB ID: 1IK3), was used as the template. Homology modeling of soybean LOX-1 was performed based on the reference protein model using Swiss model (http://swissmodel.expasy.org/) [26,27,28]. The optimized model was visualized using PyMol version 1.3 and evaluated by PDBsum (https://www.ebi.ac.uk/pdbsum/) and PROCHECK for validation of the 3D structure and stereochemical quality, respectively [29,30].

### 3.4. Molecular Docking

Docking studies were carried out using cDOCKER protocol under the receptor-ligand interaction section in Discovery Studio^®^ 3.1 (Accelrys, Inc., San Diego, CA, USA) based on scoring option. The receptor was the homology model and was pretreated before docking by adding hydrogen atoms, and all ionizable residues were set at their default protonation of pH 7.4. Meanwhile, all the 3D structures of the ligands were built with ChemBioOffice^®^ 2008 (PerkinElmer, Inc., Waltham, MA, USA) and minimized. The receptor was held rigid while the ligands were allowed to flex during the docking process. The heating and cooling temperature were set, respectively, to 700 K in 2000 steps and 300 K in 5000 steps, and the grid extension was set to 10 Å. Finally, 10 ligand-binding poses were ranked according to their cDOCKER energy, and the predicted binding interactions were analyzed [31].

### 3.5. Molecular Dynamic Simulation

The docked structure of the ligands and LOX-1 (homology model) were obtained from cDOCKER. The parameterization of ligands was carried out using the Automated Topology Builder (ATB) module implemented in the Groningen Machine for Chemical Simulation (GROMACS) 4 program [32]. The input files for MD simulations were prepared using the “grompp” script in GROMACS, and the field of united-atom GROMOS96 54a7 was chosen [33]. A 10 Å isomeric truncated octahedral box of pre-equilibrated TIP3P water was used to immerse the prepared complex structure while counterions were added to neutralize the net charge of the system [34]. To adjust the ionic strength, 0.150 M of NaCl was added.

At first, position restraints on residues 499, 504, 690, and 694 were carried out by using the steepest descent algorithm followed by energy minimization. The constant number of particles, volume, and temperature (NVT) with the coupling scheme of V-rescale and constant number of particles, pressure, and temperature (NPT) ensembles with the coupling scheme of Parrinello–Rahman were performed in association with position restraint on ligand. The parameters for NVT were 310 K with a coupling constant of 0.1 ps with duration of 1000 ps for temperature stabilization, while pressure stabilization was done by adopting a constant pressure of 1 bar with a coupling constant of 2.0 ps with duration of 1000 ps. The particle mesh Ewald (PME) method interaction was used with a 1.4 Å cutoff for short range and the Lincs algorithm for all bond constraints [35,36,37]. Simulations were performed for 10 ns with a 2 fs time step.

The evolution of structural properties over time, including the backbone atoms root mean square deviations (Cα RMSd), were calculated from the MD trajectories using GROMACS distribution tools.

MM-PBSA calculations: The binding free energies were calculated based on a single trajectory approach through the snapshots taken during MD simulation. A summation of average energy of molecular mechanics (∆E_MM_) and solvation free energy (∆G_solv_) contributed binding free energy (∆G_bind_) (1), while bonded (E_bond_, E_angle_, and E_torsion_) and nonbonded (E_vdw_ and E_EEL_) contributed average molecular mechanics energy ∆E_MM_ (2). Summation of the polar solvation free energy evaluated using the Poisson–Boltzmann equation, ∆G_PB_, and the nonpolar contribution to exterior water gave ∆G_solv_ (3). Solvent-accessible surface area (SASA) with a probe radius of 1.4 Å was estimated by using the Maximum Speed Molecular Surface (MSMS) algorithm. The surface tension proportionality constant γ was set to 0.0072 kcal/mol/Å^2^ to calculate total nonpolar solvation energy [38,39]. The overall equations are summarized as follows:

∆G = ∆E_MM_ + ∆G_solv_ (1)

In which:

∆E_MM_ = ∆E_bond_ + ∆E_angle_ + ∆E_torsion_ + ∆E_vdw_ + ∆E_EEL_ (2)

∆G_solv_ = ∆G_PB_ + ∆G_SA_ (3)

∆G_SA_ = γSA + b (4)

### 3.6. ADMET and TOPKAT Analyses

ADMET analysis and toxicity profiling (TOPKAT) of all tHGA analogues were performed using Discovery Studio^®^ 3.1 (Accelrys, Inc., San Diego, CA, USA). The ADMET analysis included aqueous solubility (AS), human intestinal absorption (HIA), blood–brain barrier (BBB), cytochrome P450 2D6 (CYP2D6), plasma protein binding (PPB), and hepatotoxicity (HT) descriptors. The toxicity prediction profile included aerobic biodegradability, mutagenicity, rodent carcinogenicity, ocular irritancy, skin irritancy, and skin sensitization descriptors.

## 4. Conclusions

In an attempt to develop a new LOX inhibitor, a series of **3a** (tHGA) analogues were synthesized and evaluated for their soybean 15-LOX inhibitory activity. It was found that substituting the geranyl chain with prenyl did not improve the inhibitory activity, and the geranylated compound **3e** with the longest aliphatic chain length on the acyl group was shown to be the most promising LOX inhibitor from the entire series. The results from the computational studies agreed well with the bioassay results. Therefore, the present study provides some useful insights into the way tHGA and its analogues bind in the active site of the soybean LOX-1 enzyme. Furthermore, ADMET and TOPKAT analyses facilitated an understanding of the drug efficiency and possible toxicity of the compounds. These findings suggest that the –OH groups of the phloroglucinol moiety, the =CO group of the acyl moiety, and the lipophilic nature of the prenylating group all contributed to the LOX inhibition. It is also suggested that lengthening the prenylating group could further improve the bioactivity and is thus recommended for future design of analogues.

## Figures and Tables

**Table 1:** IC_50_ values and % inhibition for the inhibition of soybean lipoxygenase (LOX)-1 by the tested compounds. NDGA, nordihydroguaiaretic acid.

Compound	% Inhibition (100 μg/mL)	IC_50_ Value (μM) Mean ± SEM
**3a** (tHGA) ^#^	94.3 ± 3.4	23.6 ± 1.7
**3b** ^#^	90.3 ± 4.1	27.6 ± 3.6
**3c** ^#^	94.4 ± 3.0	12.3 ± 0.6
**3d**	90.7 ± 5.0	15.3 ± 0.5
**3e** ^#^	90.3 ± 4.4	10.3 ± 1.5
**3f** ^#^	88.8 ± 5.9	26.3 ± 1.3
**3g** ^#^	90.8 ± 7.6	15.2 ± 1.2
**4a**	94.8 ± 5.2	72.1 ± 1.2
**4b**	82.5 ± 7.3	53.7 ± 0.7
**4c**	78.1 ± 3.9	61.0 ± 1.1
**4d**	86.2 ± 6.9	39.5 ± 1.2
**4e**	79.4 ± 6.7	35.1 ± 1.3
**4f**	93.7 ± 3.8	52.6 ± 1.0
**4g**	95.2 ± 3.2	95.4 ± 4.0
**NDGA**	100.0 ± 0.0	0.1 ± 0.0

^#^ Data published in previous work [13].

**Table 2:** *K_m_* (Michaelis–Menten constant), *V_max_* (maximum enzyme velocity), and *K_i_* (inhibitor constant) for the inhibition of soybean LOX-1 by the tested compounds.

Compounds	Concentrations (μg/mL)						*K_i_* (μM)
	0		1.57		3.13		
	*K_m_*	*V_max_*	*K_m_*	*V_max_*	*K_m_*	*V_max_*	
**3e**	4.62	0.035	5.20	0.035	5.78	0.035	36.1
tHGA (**3a**)	2.63	0.020	2.96	0.020	3.29	0.020	41.1

**Table 3:** Summary of binding free energy (∆G_bind_) for compounds **3e**, **4e** and tHGA (**3a**).

Compound	Van der Waals Energy	Electrostatic Energy	Polar Solvation Energy	Nonpolar Solvation Energy	Total Binding Energy
**3e**	−200.44 (8.75)	−16.93 (8.53)	87.65 (13.26)	−20.99 (1.02)	−150.71 (11.30)
**4e**	−175.29 (7.67)	−7.39 (5.74)	89.10 (11.91)	−18.21 (0.99)	−111.79 (12.59)
tHGA (**3a**)	−171.62 (9.16)	−14.40 (4.19)	57.28 (9.08)	−19.30 (0.93)	−148.04 (10.89)

**Table 4:** Results of absorption, distribution, metabolism, excretion, and toxicity (ADMET) prediction on selected parameters of selected compounds.

Compound	HIA	AS	BBB	PBB	CYP2D6	HT
**3a** **	Good	Good	Medium	Bound	Inhibitor	Nontoxic
**3b**	Moderate	Low	–	Bound	Inhibitor	Nontoxic
**3c**	Moderate	Low	–	Bound	Inhibitor	Nontoxic
**3d**	Moderate	Low	–	Bound	Inhibitor	Nontoxic
**3e** *	Poor	Low	–	Bound	Inhibitor	Nontoxic
**3f**	Poor	Low	–	Bound	Noninhibitor	Nontoxic
**3g**	Poor	Low	–	Bound	Noninhibitor	Nontoxic
**4a**	Good	Good	Good	Bound	Noninhibitor	Toxic
**4b**	Good	Good	Medium	Bound	Noninhibitor	Toxic
**4c**	Good	Good	Medium	Bound	Noninhibitor	Toxic
**4d**	Good	Good	Medium	Bound	Inhibitor	Toxic
**4e**	Good	Good	Medium	Bound	Inhibitor	Toxic
**4f**	Good	Low	High	Bound	Noninhibitor	Toxic
**4g**	Good	Low	High	Bound	Noninhibitor	Toxic

* Most active compound; ** parent compound. HIA, human intestinal absorption; AS, aqueous solubility; BBB, blood–brain barrier; PPB, protein plasma binding; CYP2D6, cytochrome P450 2D6; HT, hepatotoxicity.

**Table 5:** Results of toxicity predictive test on six important parameters.

Compound	AB	AM	RC	OI	SI	SS
**3a** **	Degradable	Nonmutagen	Noncarcinogen	Nonirritant	Nonirritant	Sensitizer
**3b**	Degradable	Nonmutagen	Noncarcinogen	Nonirritant	Nonirritant	Sensitizer
**3c**	Degradable	Nonmutagen	Noncarcinogen	Nonirritant	Nonirritant	Sensitizer
**3d**	Degradable	Nonmutagen	Noncarcinogen	Nonirritant	Nonirritant	Sensitizer
**3e** *	Degradable	Nonmutagen	Noncarcinogen	Nonirritant	Nonirritant	Sensitizer
**3f**	Degradable	Nonmutagen	Noncarcinogen	Nonirritant	Nonirritant	Sensitizer
**3g**	Degradable	Nonmutagen	Noncarcinogen	Nonirritant	Nonirritant	Sensitizer
**4a**	Degradable	Nonmutagen	Noncarcinogen	Nonirritant	Nonirritant	Sensitizer
**4b**	Degradable	Nonmutagen	Noncarcinogen	Irritant	Nonirritant	Sensitizer
**4c**	Degradable	Nonmutagen	Noncarcinogen	Irritant	Nonirritant	Sensitizer
**4d**	Degradable	Nonmutagen	Noncarcinogen	Irritant	Nonirritant	Sensitizer
**4e**	Degradable	Nonmutagen	Noncarcinogen	Irritant	Nonirritant	Sensitizer
**4f**	Degradable	Nonmutagen	Noncarcinogen	Irritant	Nonirritant	Sensitizer
**4g**	Nondegradable	Nonmutagen	Noncarcinogen	Nonirritant	Nonirritant	Sensitizer

* Most active compound; ** parent compound. AB, aerobic biodegradability; AM, Ames mutagenicity; RC, rodent carcinogenicity; OI, ocular irritancy; SI, skin irritancy; SS, skin sensitization.

## Supplementary Materials

The following are available online: Full experimental data (including ^1^H, ^13^C NMR, ESI-MS) for all synthetic compounds are provided. Additionally, the computational work (including sequence alignment, homology modeling, and molecular dynamic simulation) is also provided.
