# Supplementary material for: Hits-to-Lead Optimization of the Natural Compound 2,4,6-Trihydroxy-3-geranyl-acetophenone (tHGA) as a Potent LOX Inhibitor: Synthesis, Structure-Activity Relationship (SAR) Study, and Computational Assignment
Source: Molecules. 2018 Sep 30;23(10):2509. doi: 10.3390/molecules23102509 (PMC6222424; doi:10.3390/molecules23102509)
Supplement: Supplementary file 1 [file molecules-23-02509-s001.pdf]

Supplementary Data

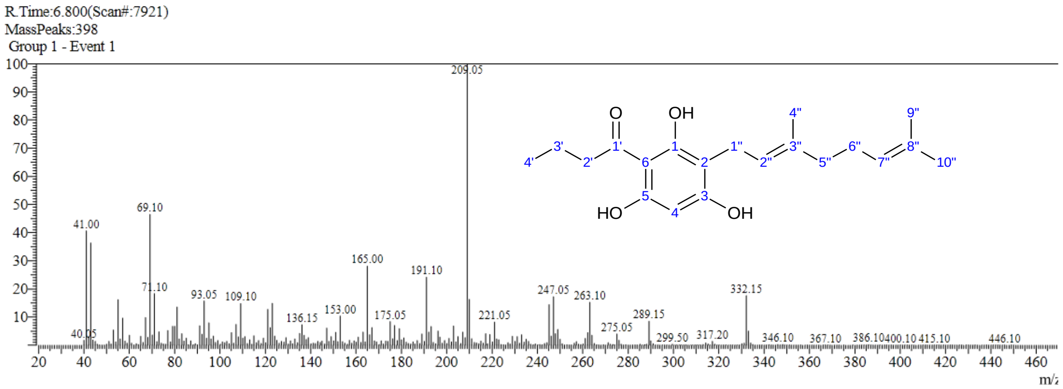

Figure S1: EIMS spectrum for *(E)-1-(3-(3,7-dimethylocta-2,6-dienyl)-2,4,6-trihydroxyphenyl)butan-1-one* (3d)

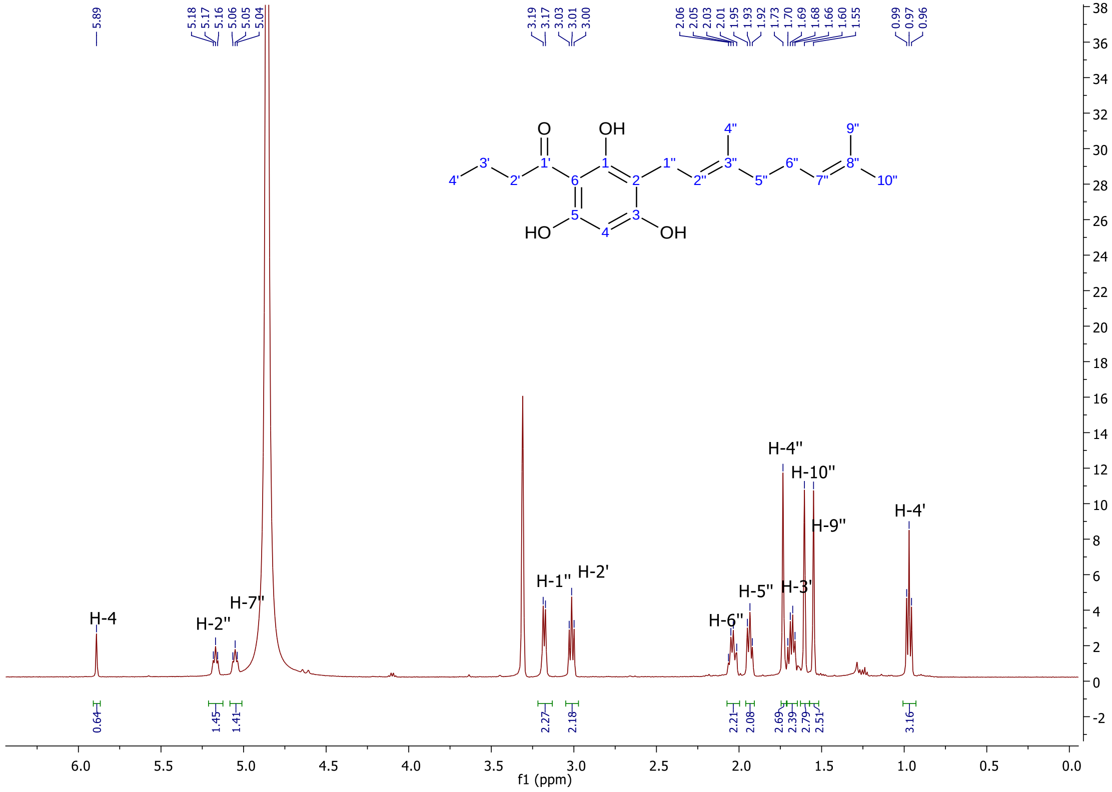

Figure S2: ^{1}H NMR spectrum for *(E)-1-(3-(3,7-dimethylocta-2,6-dienyl)-2,4,6-trihydroxyphenyl)butan-1-one* (3d)

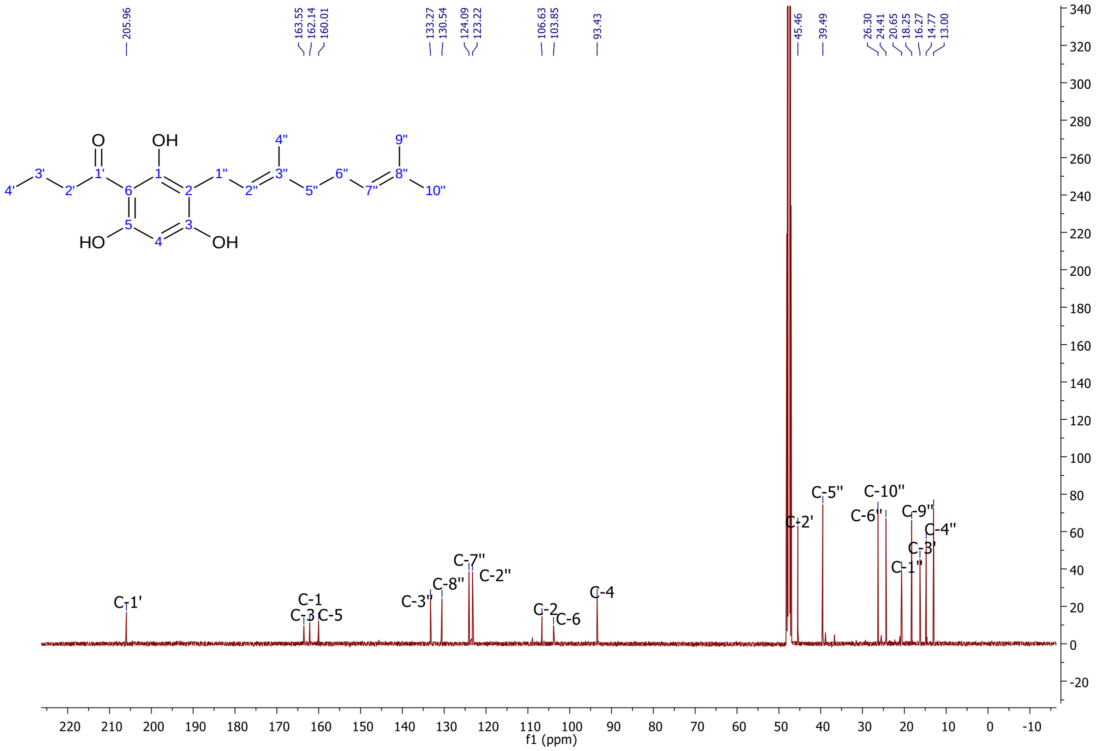

Figure S3: ^{13}C NMR spectrum for *(E)-1-(3-(3,7-dimethylocta-2,6-dienyl)-2,4,6-trihydroxyphenyl)butan-1-one* (3d)

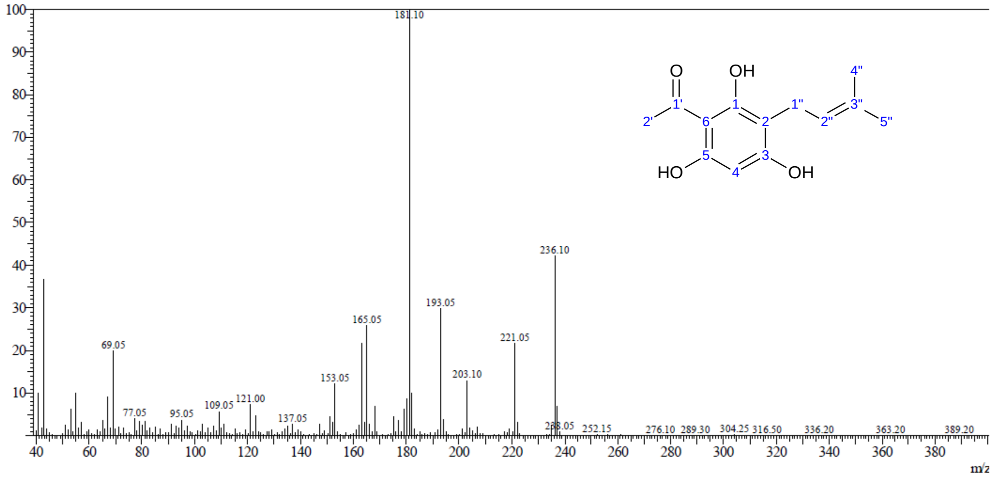

Figure S4: EIMS spectrum for *1-(2,4,6-trihydroxy-3-(3-methylbut-2-enyl)phenyl)ethanone* (4a)

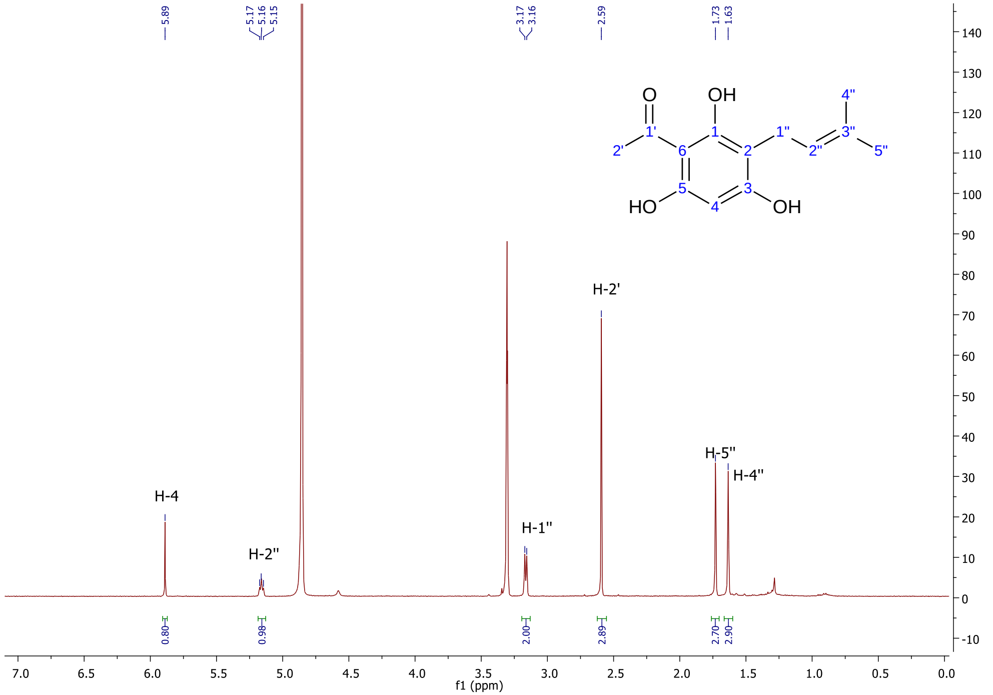

Figure S5: ^{1}H NMR spectrum for *1-(2,4,6-trihydroxy-3-(3-methylbut-2-enyl)phenyl)ethanone* (4a)

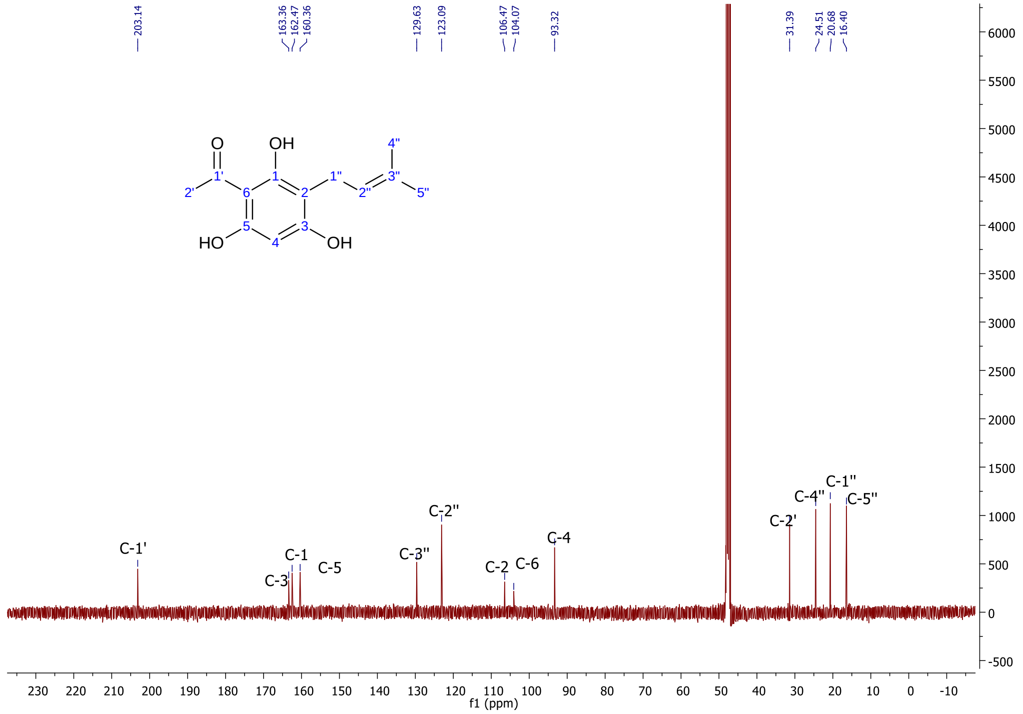

Figure S6: ^{13}C NMR spectrum for *1-(2,4,6-trihydroxy-3-(3-methylbut-2-enyl)phenyl)ethanone* (4a)

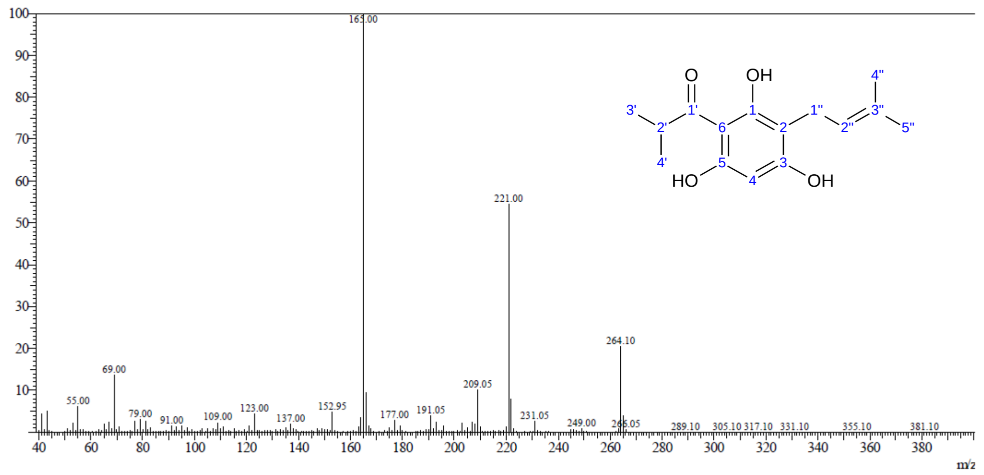

Figure S7: EIMS spectrum for *2-methyl-1-(2,4,6-trihydroxy-3-(3-methylbut-2-enyl)phenyl)propan-1-one* (4b)

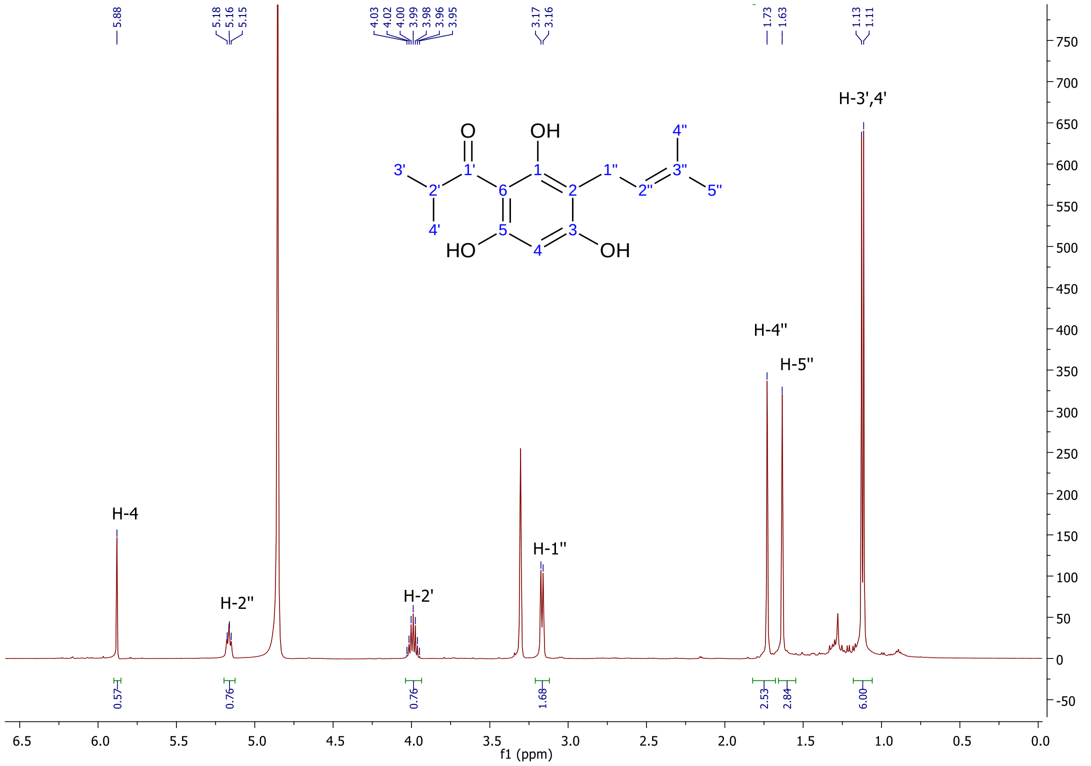

Figure S8: ¹H NMR spectrum for *2-methyl-1-(2,4,6-trihydroxy-3-(3-methylbut-2-enyl)phenyl)propan-1-one* (4b)

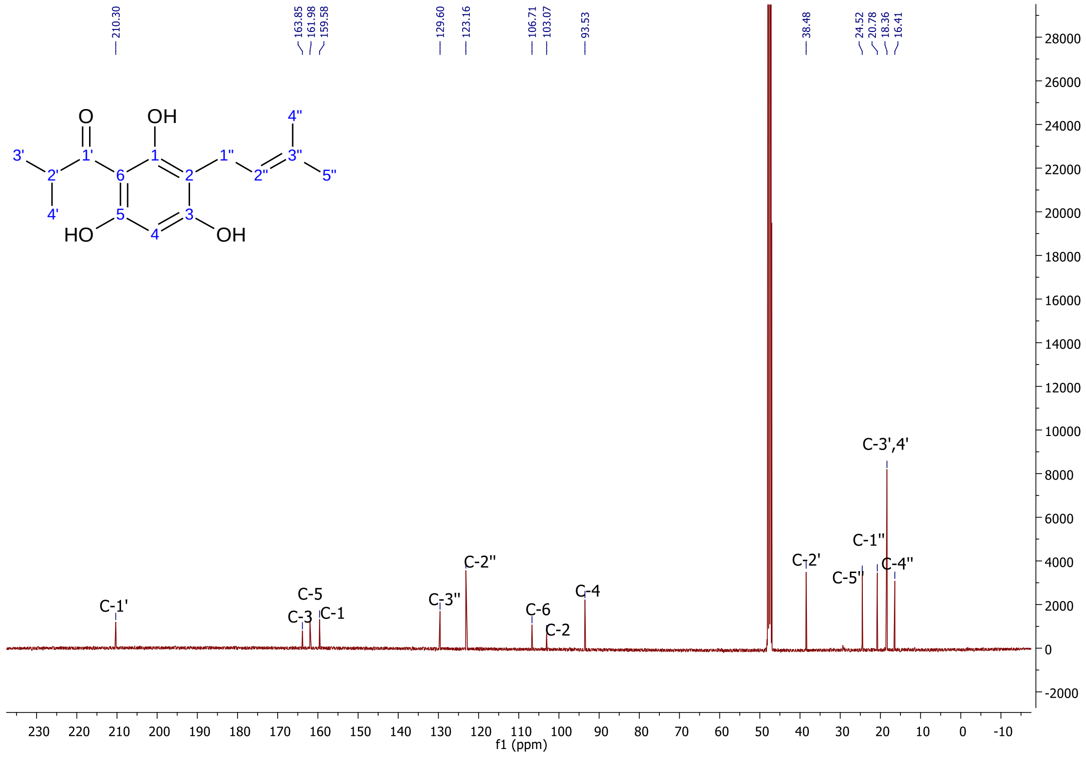

Figure S9: ^{13}C NMR spectrum for *2-methyl-1-(2,4,6-trihydroxy-3-(3-methylbut-2-enyl)phenyl)propan-1-one* (4b)

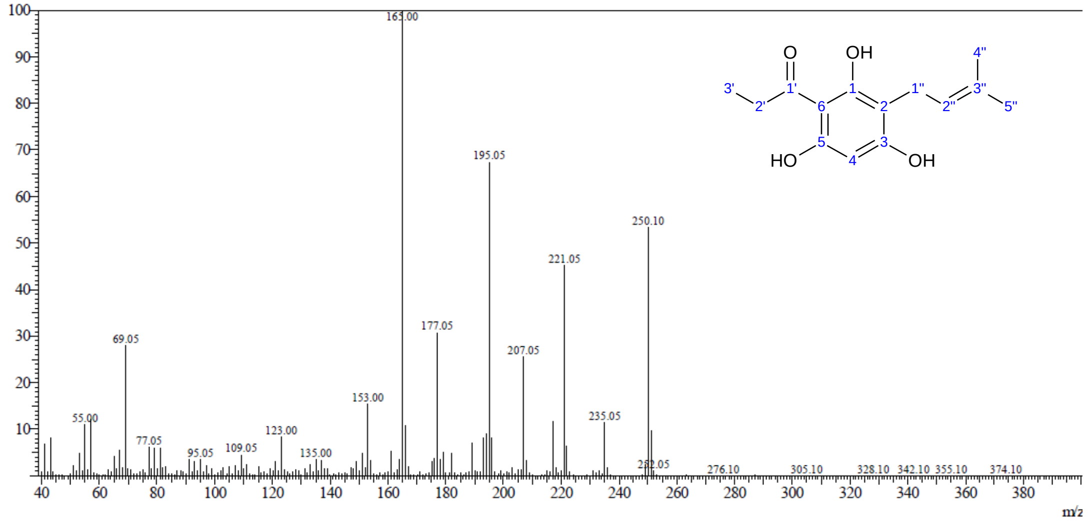

Figure S10: EIMS spectrum for *1-(2,4,6-trihydroxy-3-(3-methylbut-2-enyl)phenyl)propan-1-one* (4c)

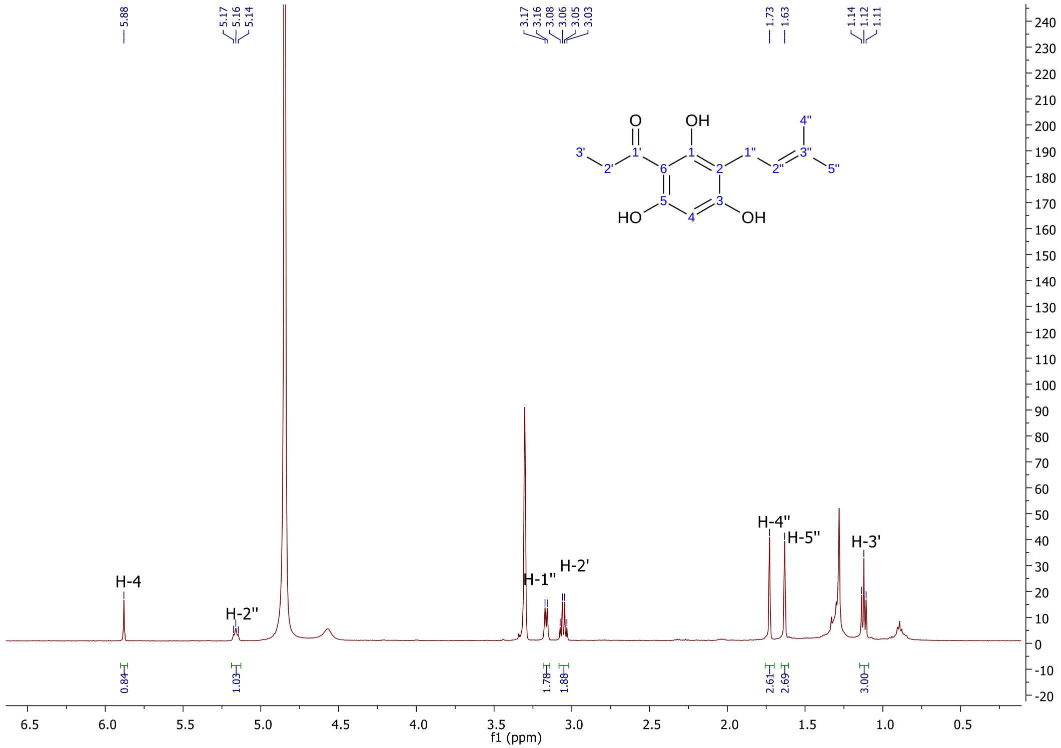

Figure S11: ^{1}H NMR spectrum for *1-(2,4,6-trihydroxy-3-(3-methylbut-2-enyl)phenyl)propan-1-one* (4c)

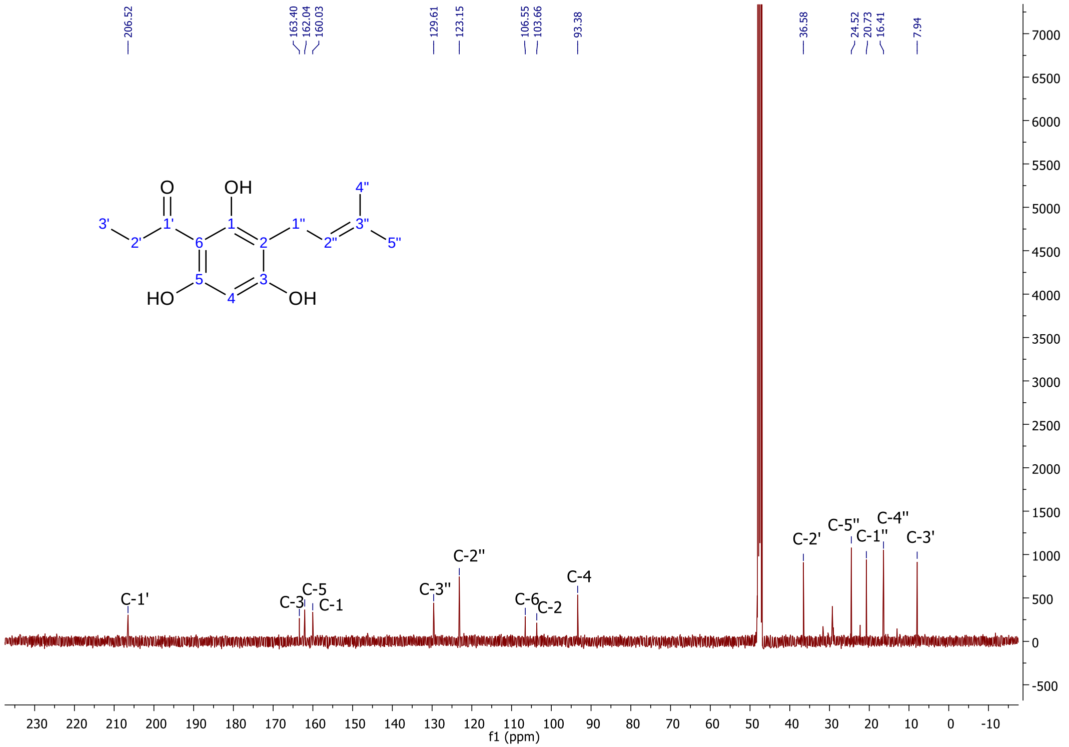

Figure S12: ^{13}C NMR spectrum for *1-(2,4,6-trihydroxy-3-(3-methylbut-2-enyl)phenyl)propan-1-one* (4c)

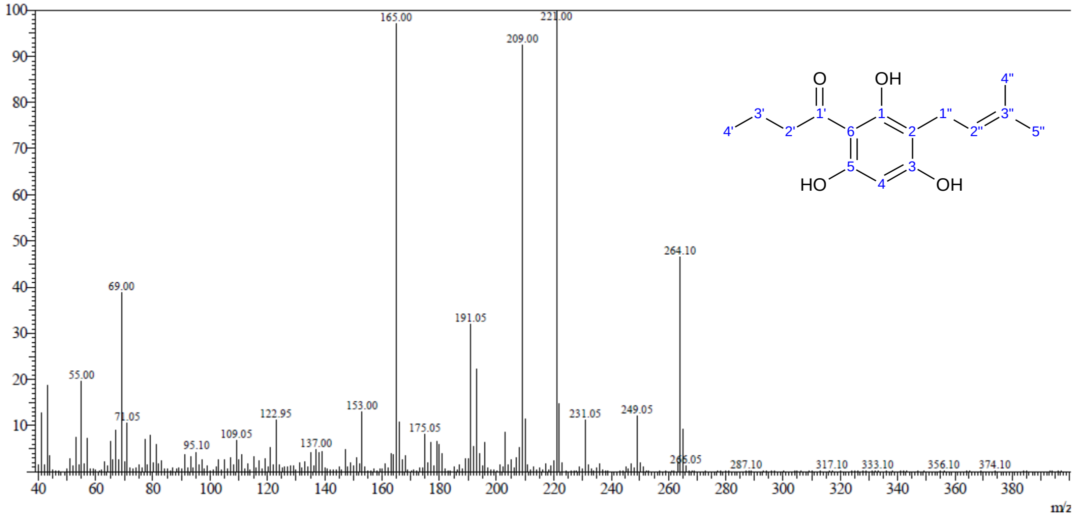

Figure S13: EIMS spectrum for *1-(2,4,6-trihydroxy-3-(3-methylbut-2-enyl)phenyl)butan-1-one* (4d)

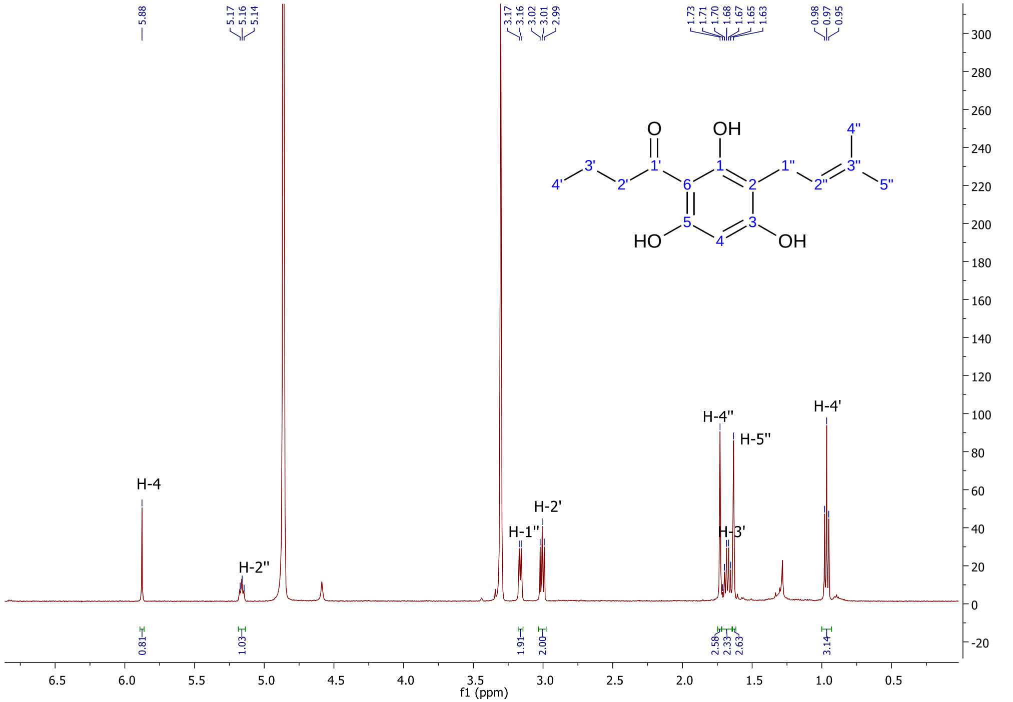

Figure S14: ^{1}H NMR spectrum for *1-(2,4,6-trihydroxy-3-(3-methylbut-2-enyl)phenyl)butan -1-one* (4d)

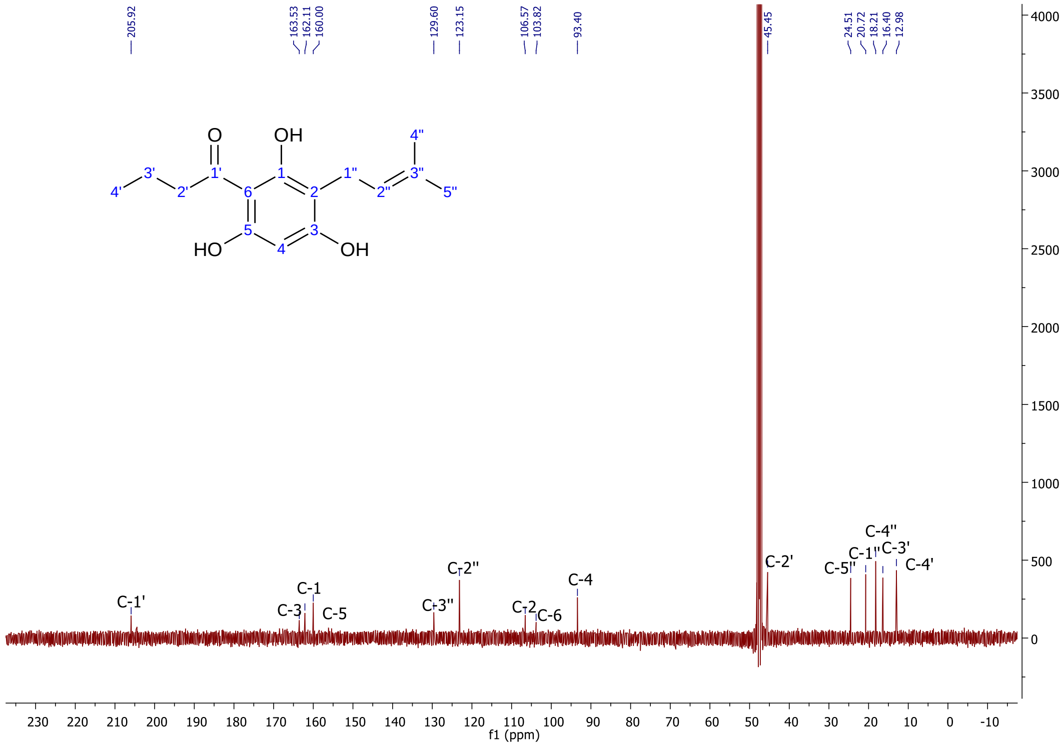

Figure S15: ^{13}C NMR spectrum for *1-(2,4,6-trihydroxy-3-(3-methylbut-2-enyl)phenyl)butan -1-one* (4d)

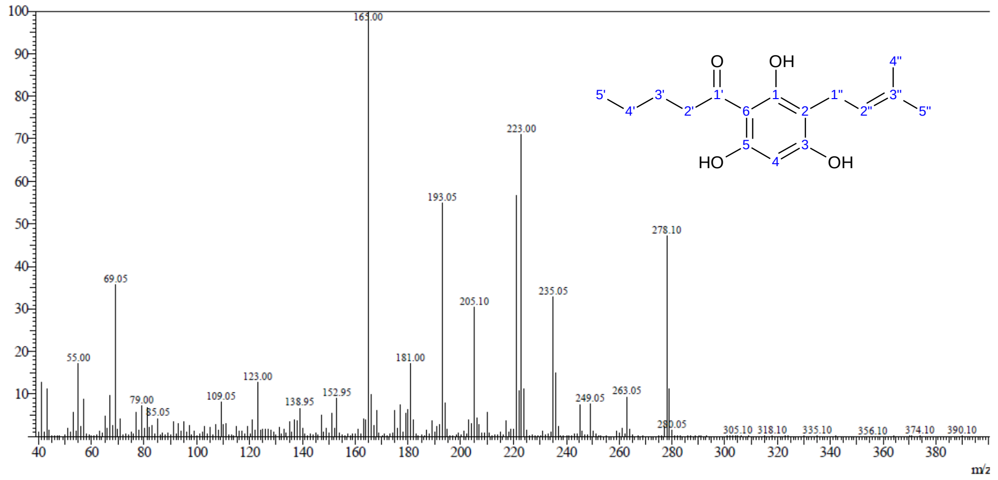

Figure S16: EIMS spectrum for *1-(2,4,6-trihydroxy-3-(3-methylbut-2-enyl)phenyl)pentan-1-one* (4e)

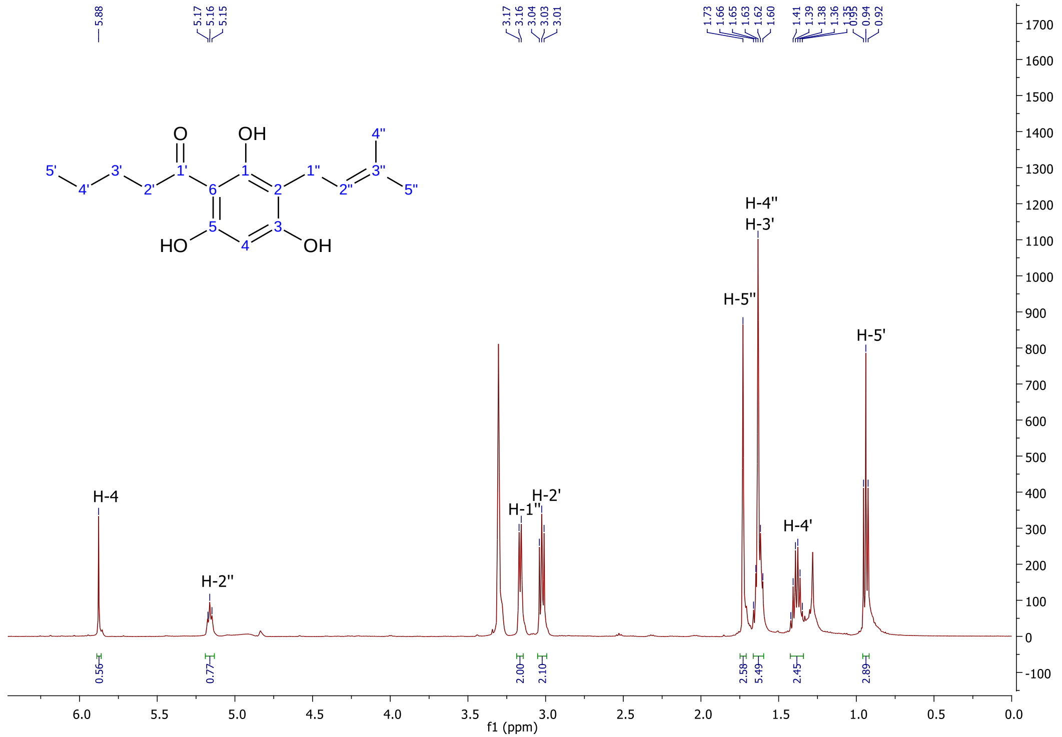

Figure S17: ^{1}H NMR spectrum for *1-(2,4,6-trihydroxy-3-(3-methylbut-2-enyl)phenyl)pentan-1-one* (4e)

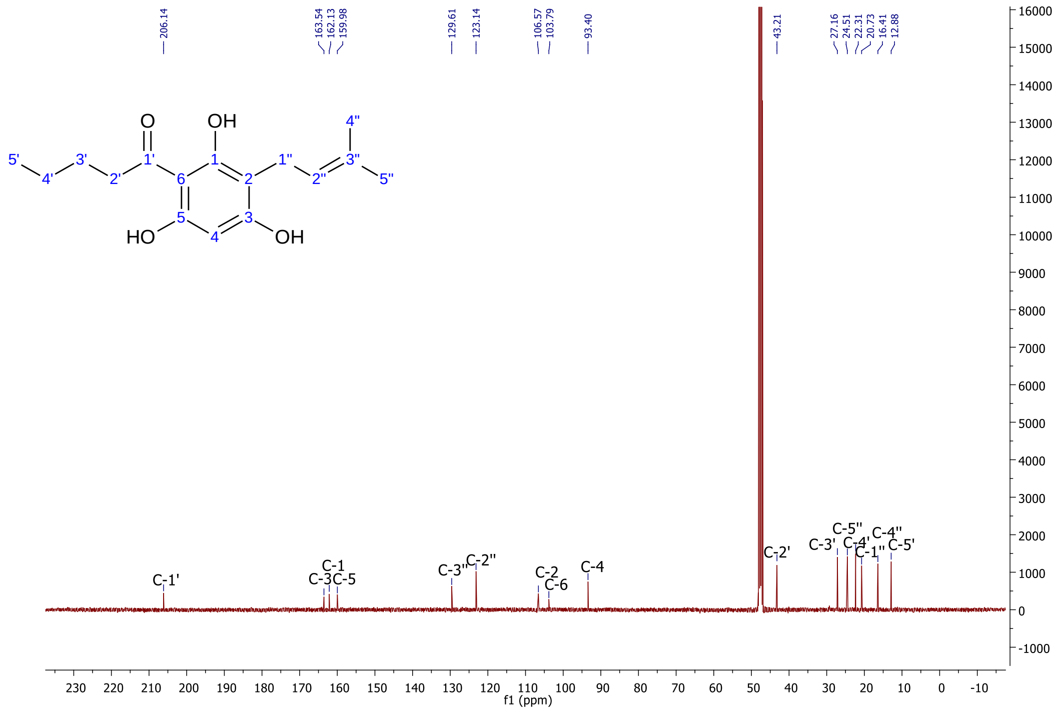

Figure S18: ^{13}C NMR spectrum for *1-(2,4,6-trihydroxy-3-(3-methylbut-2-enyl)phenyl)pentan-1-one* (4e)

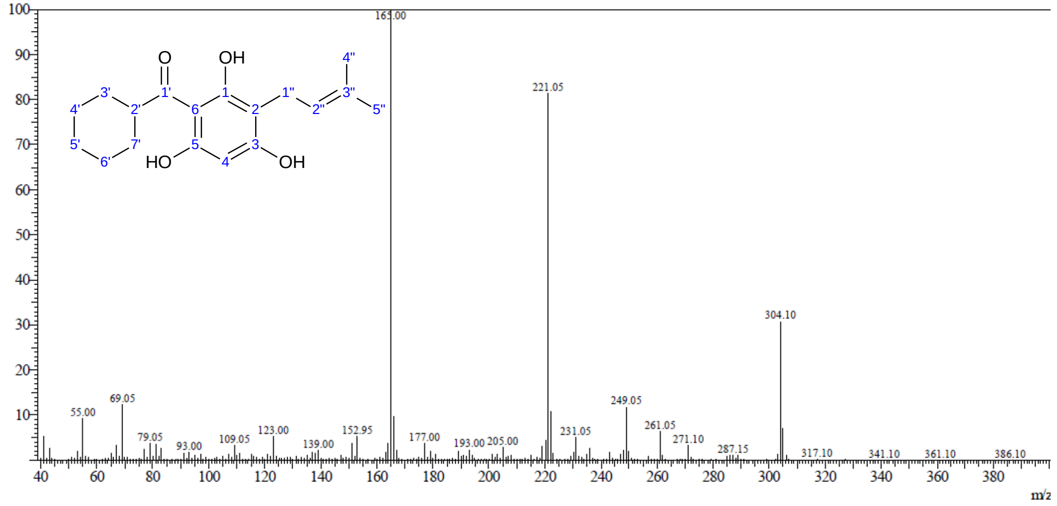

Figure S19: EIMS spectrum for *Cyclohexyl-(2,4,6-trihydroxy-3-(3-methylbut-2-enyl)phenyl)methanone* (4f)

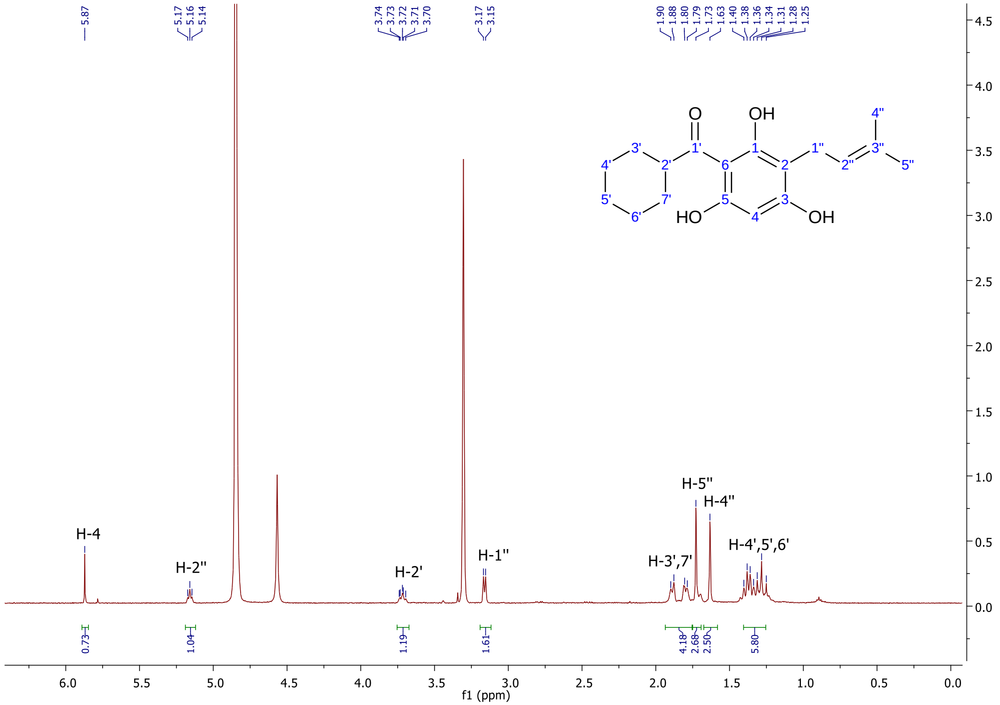

Figure S20: ^{1}H NMR spectrum for *Cyclohexyl-(2,4,6-trihydroxy-3-(3-methylbut-2-enyl)phenyl)methanone* (4f)

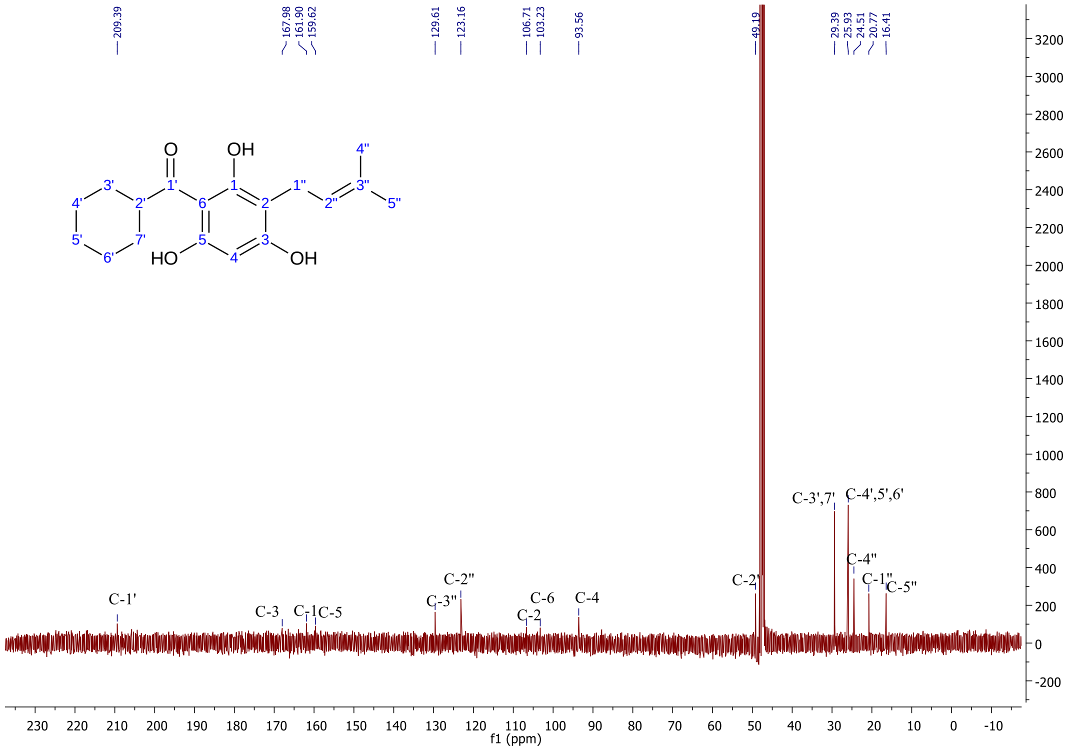

Figure S21: ^{13}C NMR spectrum for *Cyclohexyl-(2,4,6-trihydroxy-3-(3-methylbut-2-enyl)phenyl)methanone* (4f)

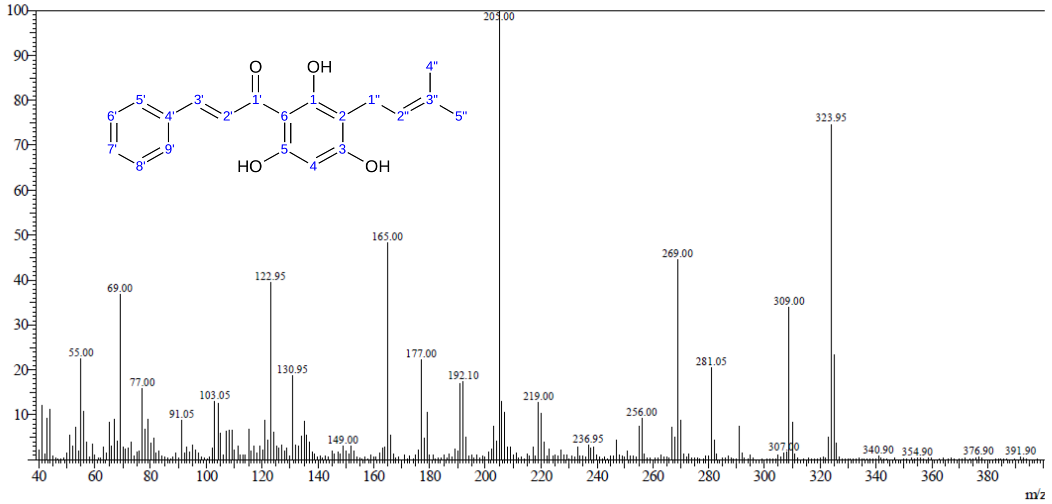

Figure S22: EIMS spectrum for *(E)-3-phenyl-1-(2,4,6-trihydroxy-3-(3-methylbut-2-enyl)phenyl)prop-2-en-1-one* (4g)

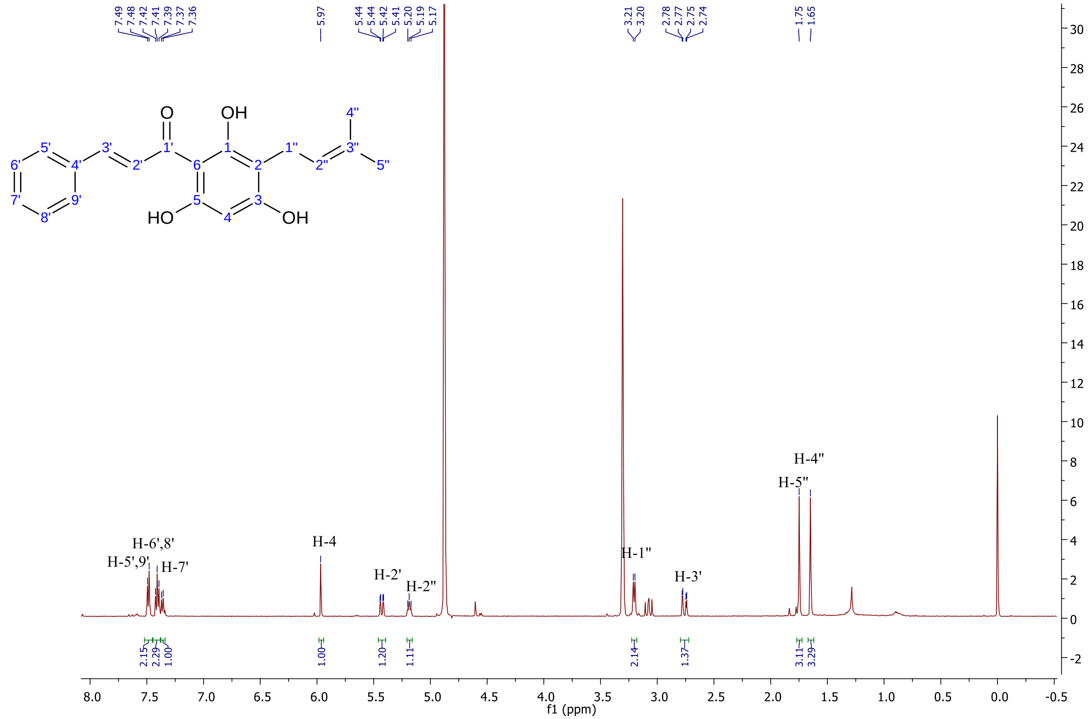

Figure S23: ^{1}H NMR spectrum for *(E)-3-phenyl-1-(2,4,6-trihydroxy-3-(3-methylbut-2-enyl)phenyl)prop-2-en-1-one* (4g)

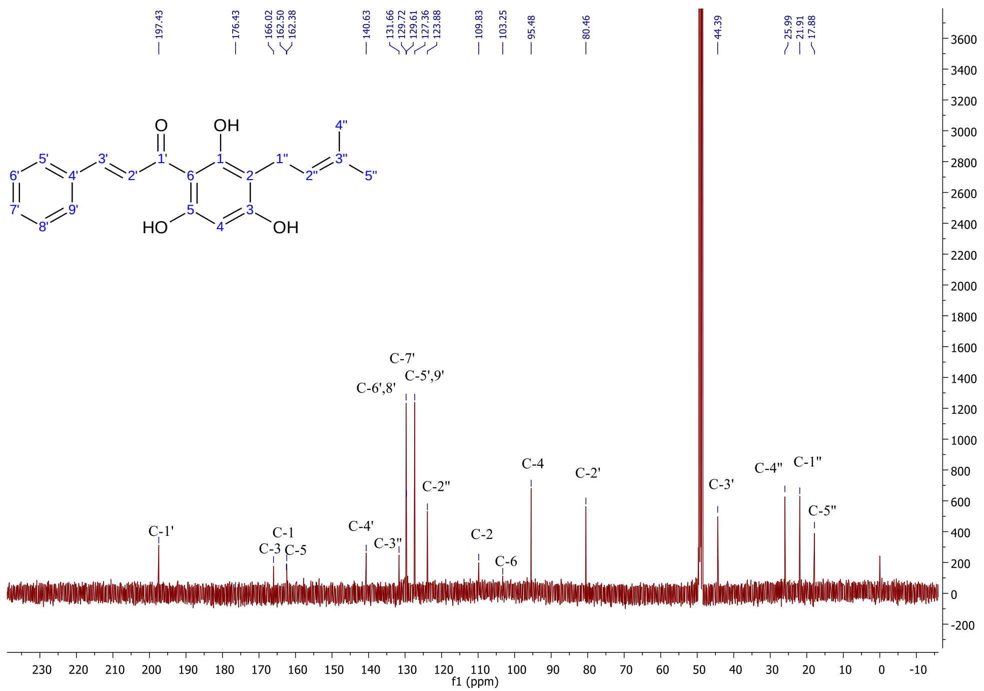

Figure S24: ^{13}C NMR spectrum for *(E)-3-phenyl-1-(2,4,6-trihydroxy-3-(3-methylbut-2-enyl)phenyl)prop-2-en-1-one* (4g)

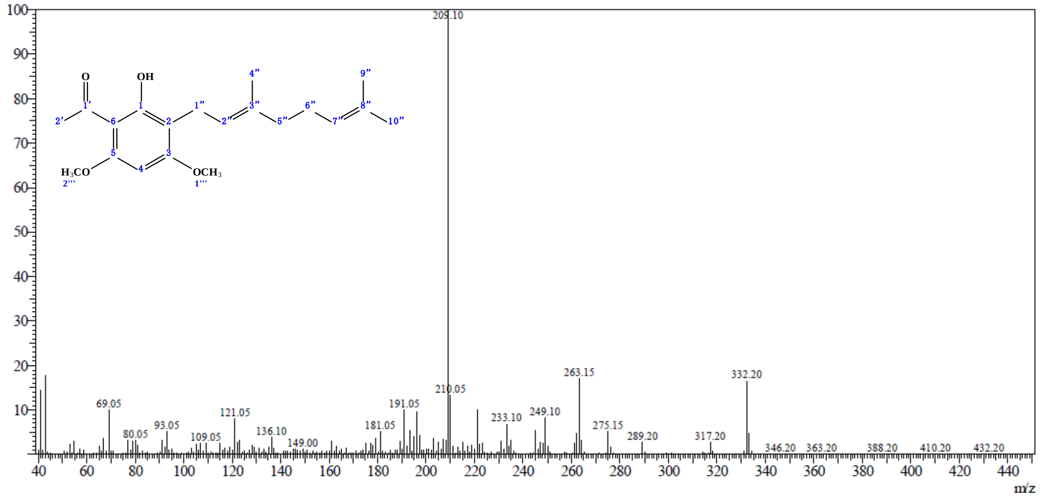

Figure S28: EIMS spectrum for *(E)-1-(3-(3,7-dimethylocta-2,6-dienyl)-2-hydroxy-4,6-dimethoxyphenyl)ethanone* (5a)

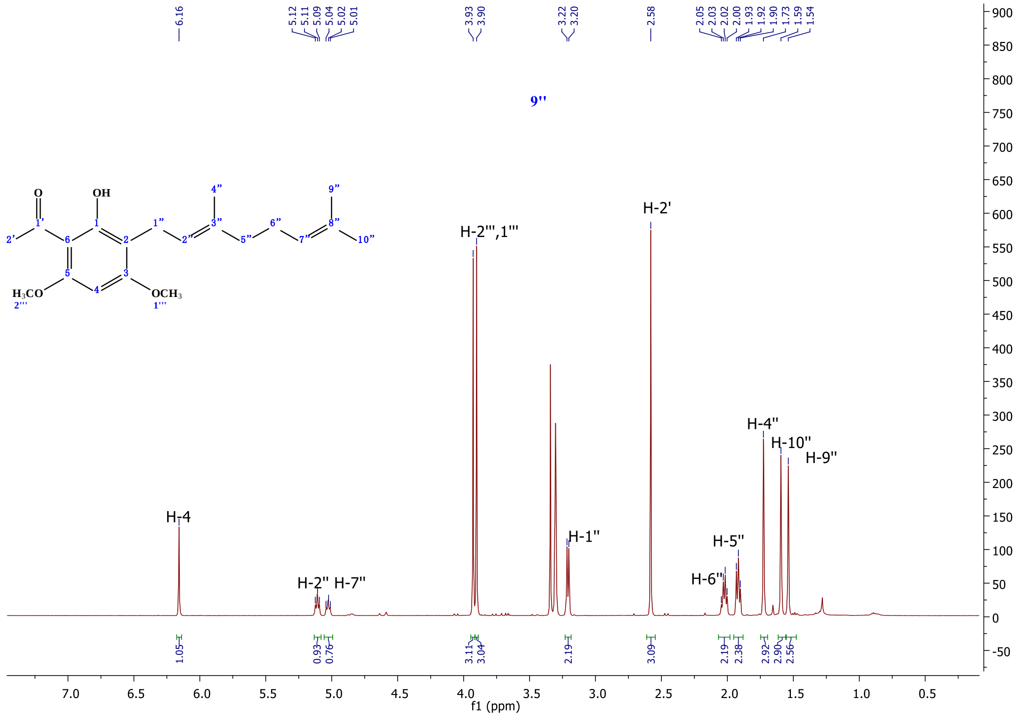

Figure S29: ^{1}H NMR spectrum for *(E)-1-(3-(3,7-dimethylocta-2,6-dienyl)-2-hydroxy-4,6-dimethoxyphenyl)ethanone* (5a)

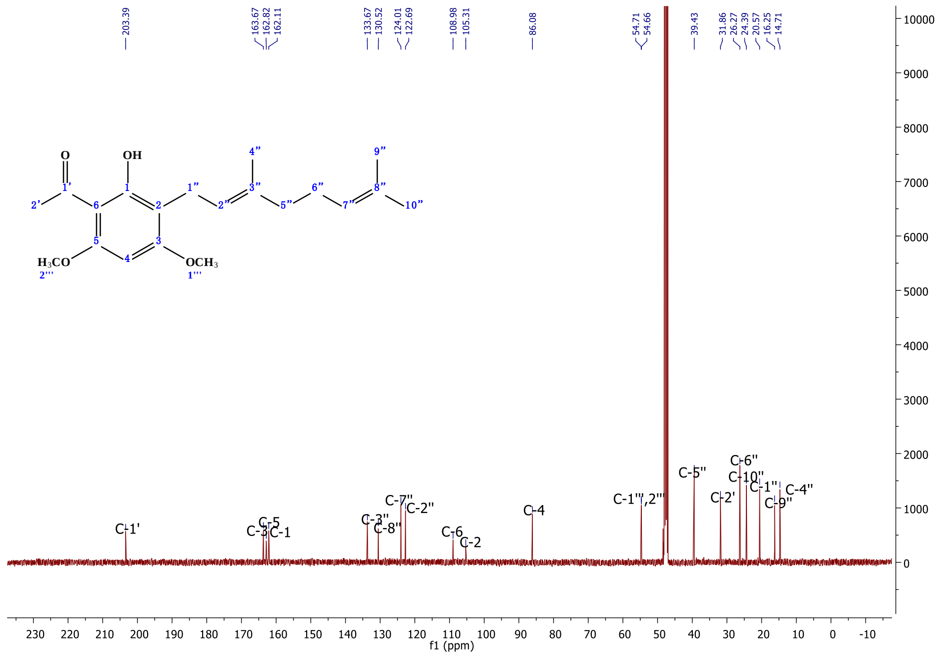

Figure S30: ^{13}C NMR spectrum for *(E)-1-(3-(3,7-dimethylocta-2,6-dienyl)-2-hydroxy-4,6-dimethoxyphenyl)ethanone* (5a)

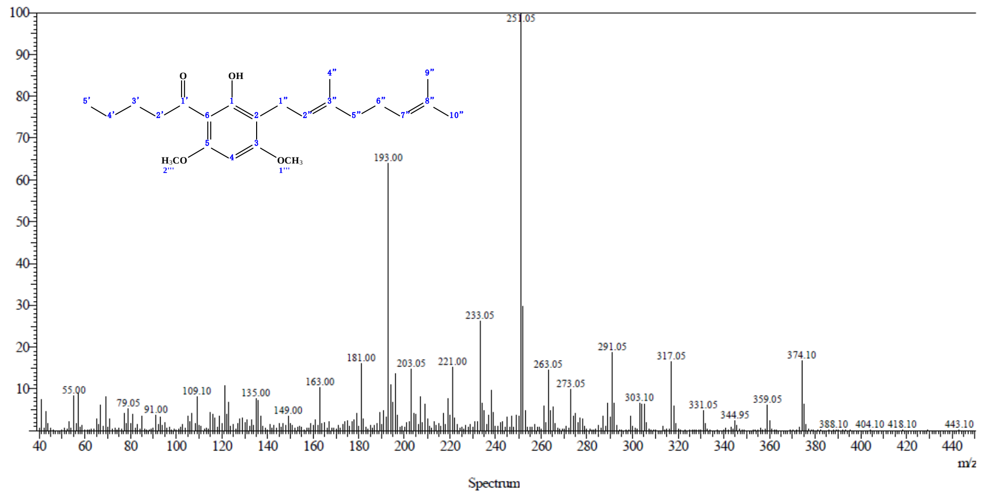

Figure S25: EIMS spectrum for *(E)-1-(3-(3,7-dimethylocta-2,6-dienyl)-2-hydroxy-4,6-dimethoxyphenyl)pentan-1-one* (5e)

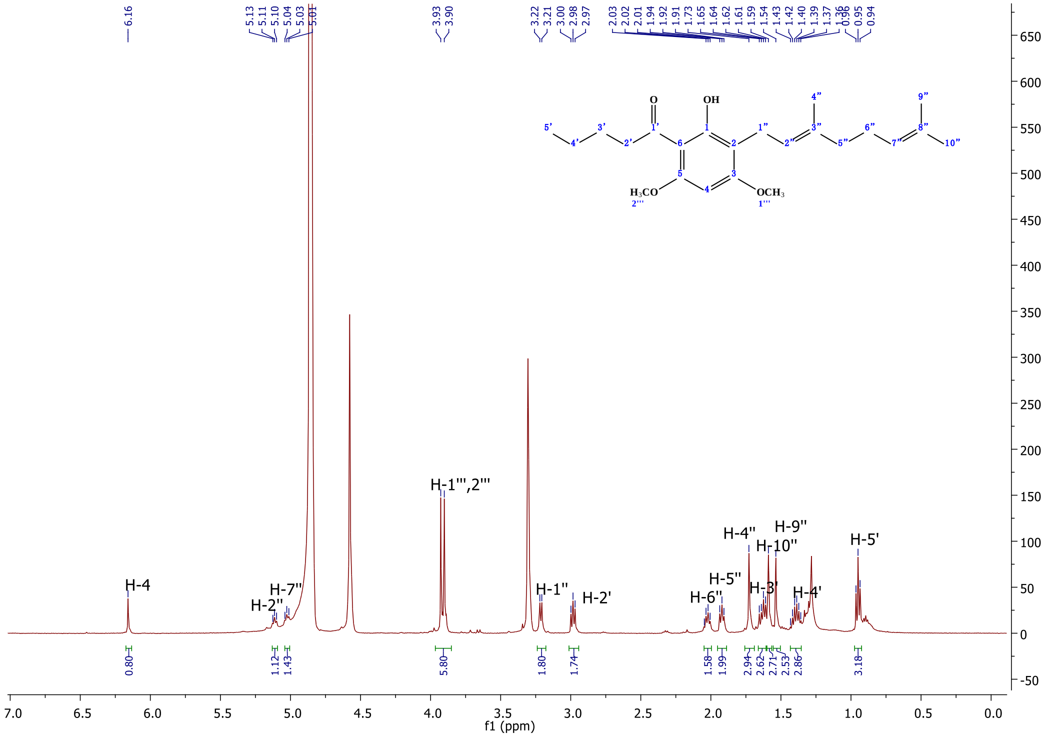

Figure S26: ^{1}H NMR spectrum for *(E)-1-(3-(3,7-dimethylocta-2,6-dienyl)-2-hydroxy-4,6-dimethoxyphenyl)pentan-1-one* (5e)

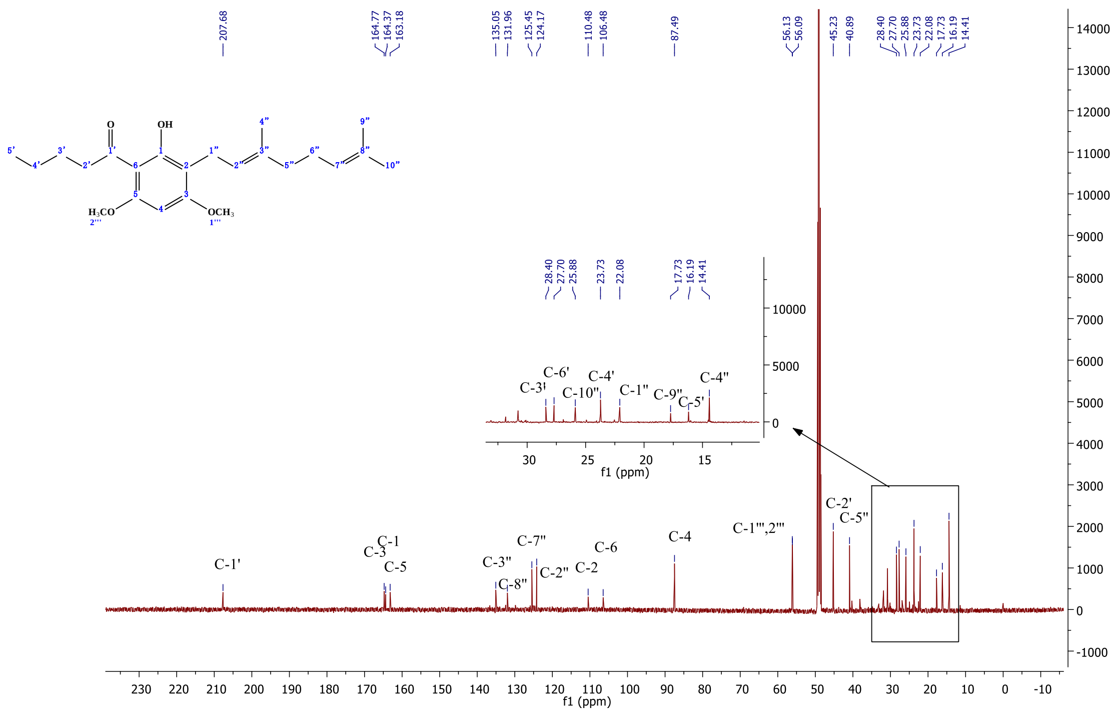

Figure S27: ^{13}C NMR spectrum for *(E)-1-(3-(3,7-dimethylocta-2,6-dienyl)-2-hydroxy-4,6-dimethoxyphenyl)pentan-1-one* (5e)

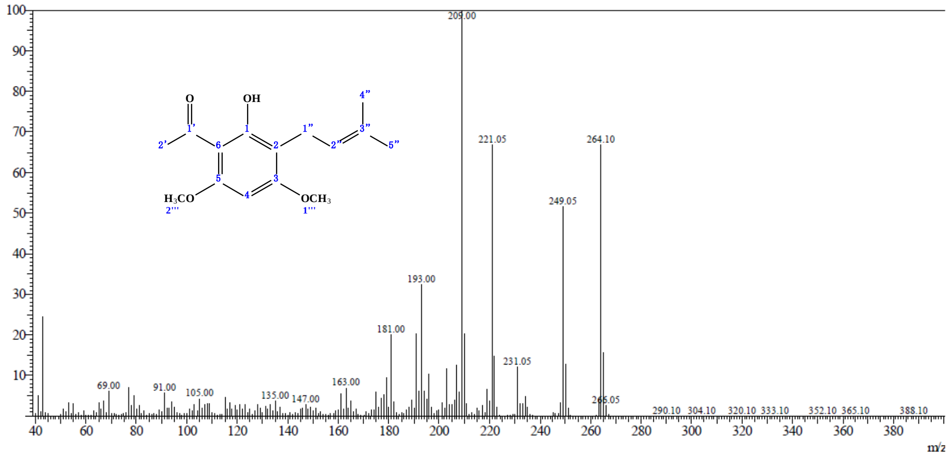

Figure S34: EIMS spectrum for *1-(2-hydroxy-4,6-dimethoxy-3-(3-methylbut-2-enyl)phenyl)ethanone* (6a)

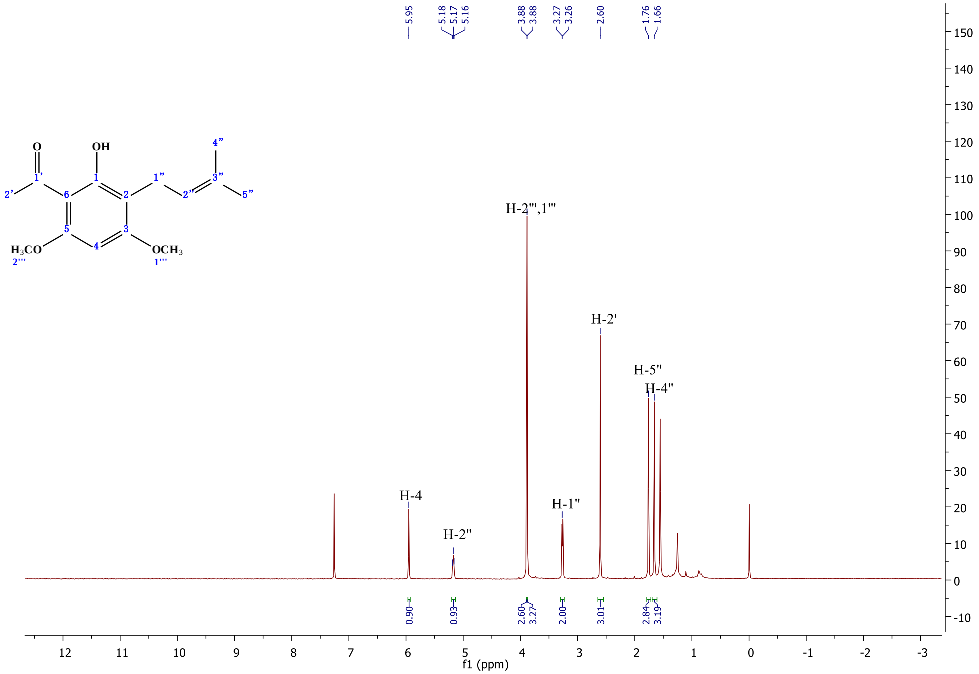

Figure S35: ^{1}H NMR spectrum for *1-(2-hydroxy-4,6-dimethoxy-3-(3-methylbut-2-enyl)phenyl)ethanone* (6a)

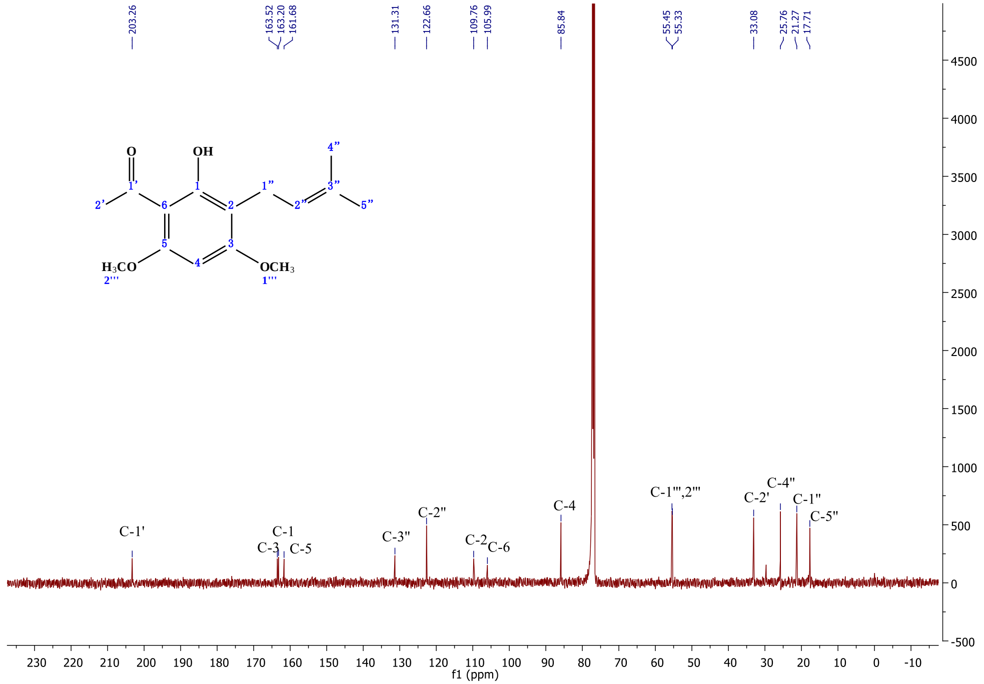

Figure S36: ^{13}C NMR spectrum for *1-(2-hydroxy-4,6-dimethoxy-3-(3-methylbut-2-enyl)phenyl)ethanone* (6a)

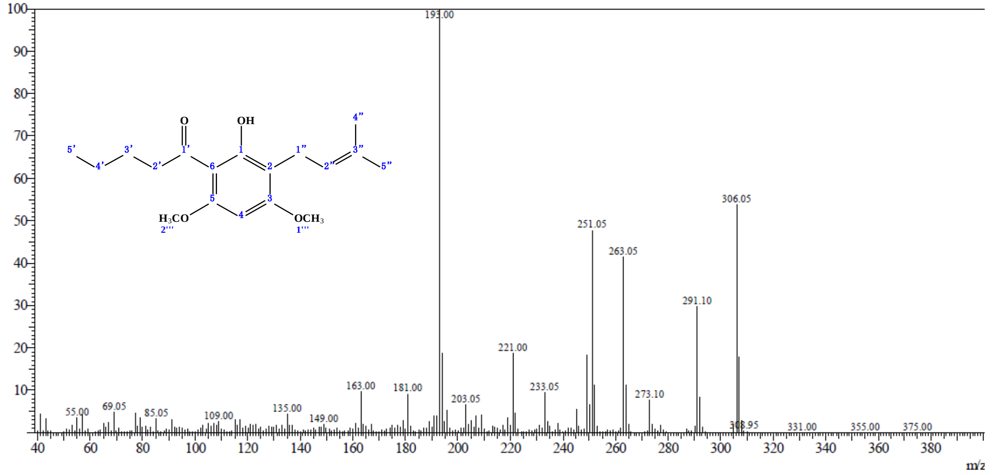

Figure S31: EIMS spectrum for *1-(2-hydroxy-4,6-dimethoxy-3-(3-methylbut-2-enyl)phenyl)pentan-1-one* (6e)

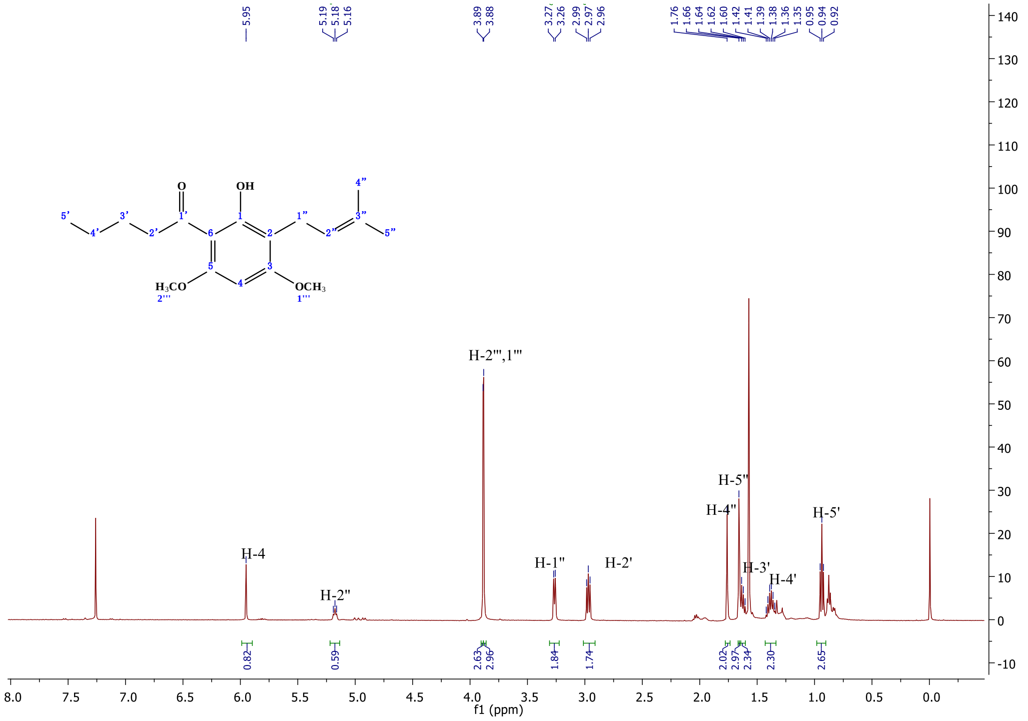

Figure S32: ^{1}H NMR spectrum for 1-(2-hydroxy-4,6-dimethoxy-3-(3-methylbut-2-enyl)phenyl)pentan-1-one (6e)

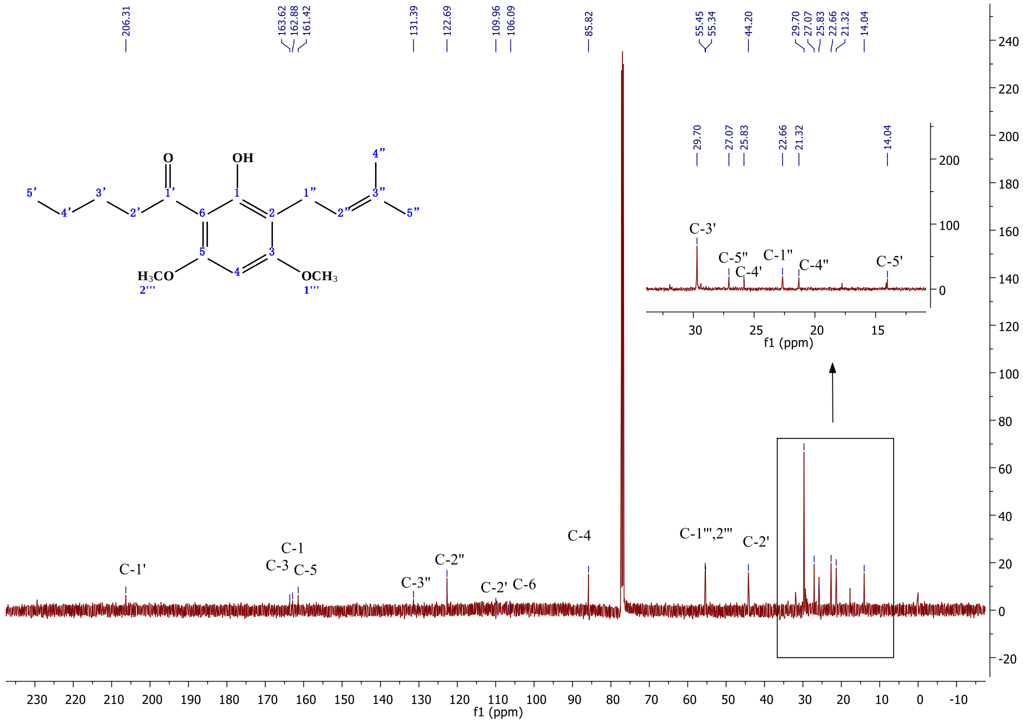

Figure S33: ^{13}C NMR spectrum for *1-(2-hydroxy-4,6-dimethoxy-3-(3-methylbut-2-enyl)phenyl)pentan-1-one* (6e)

```
LOX-1  1   MFSAGHKIKGTVVLMPKNELEVNPDGS-AVDNLNAFLGRSVSLQLISATKADAHG   54
1IK3   5   ----GHKIKGTVVLMRKNVLDVNSVTSVTLDTLTAFLGRSVSLQLISATKADANG   55

LOX-1 55   KGKVGKDTFLEGINTSLPTLGAGESAFNIHFEW-DGSMGIPGAFYIKNYMQVEFF 108
1IK3  56   KGKLGKATFLEGIITSLPTLGAGQSAFKINFEWDDGS-GIPGAFYIKNFMQTEFF 105

LOX-1 109 LKSLTLEAISNQGTIRFVCNSWVYNTKLYKSVRIFFANHTYVPSETPAPLVSYRE 163
1IK3  106 LVSLTLEDIPNHGSIHFVCNSWIYNAKLFKSDRIFFANQTYLPSETPAPLVKYRE 160

LOX-1 164 EELKSLRGNGTGERKEYDRIYDYDVYNDLGNPDKSEKLARPVLGGSSTFPYPRRG 218
1IK3  161 EELHNLRGDGTGERKEWERIYDYDVYNDLGDPDKGENHARPVLGGNDTFPYPRRG 215

LOX-1 219 RTGRGPTVTDPNTE-KQGEVFYVPRDENLGHLKSKDALEIGTKSLSQIVQPAFES 272
1IK3  216 RTGRKPTRKDPNSESRSNDV-YLPRDEAFGHLKSSDFLTYGLKSVSQNVLPLLQS 269

LOX-1 273 AFDLKSTPIEFHSFQDVHDLYEGGIKLPRDVISTIIPLPVIKELYRTDGQHILKF 327
1IK3  270 AFDLNFTPREFDSFDEVHGLYSGGIKLPTDIISKISPLPVLKEIFRTDGEQALKF 324

LOX-1 328 PQPHVVQVSQSAWMTDEEFAREMIAGVNPCVIRGLEEFPPKSNLDPAIYGDQSSK 382
1IK3  380 PPPKVIQVSKSAWMTDEEFAREMLAGVNPNLIRCLKDFPPRSKLDSQVYGDHTSQ 379

LOX-1 383 ITADSLD--LDGYTMDEALGSRRLFMLDYHDIFMPYVRQINQLNSAKTYATRTIL 435
1IK3  380 ITKEHLEPNLEGLTVDEAIQNKRLFLLDHHDPIMPYLRRIN-ATSTKAYATRTIL 433

LOX-1 436 FLREDGTLKPVAIELSLPHSAGDLSAAVSQVVLPAKEGVESTIWLLAKAYVIVND 490
1IK3  434 FLKNDGTLRPLAIELSLPHPQGDQSGAFSQVFLPADEGVESSIWLLAKAYVVVND 488

LOX-1 491 SCYHQLMSHWLNTHAAMEPFVIATHRHLSVLHPIYKLLTPHYRNNMNINALARQS 545
1IK3  489 SCYHQLVSHWLNTHAVVEPFIIATNRHLSVVHPIYKLLHPHYRDTMNINGLARLS 543
                                             *     *
LOX-1 546 LINANGIIETTFLPSKYSVEMSSAVYKNWVFTDQALPADLIKRGVAIKDPSTPHG 600
1IK3  544 LVNDGGVIEQTFLWGRYSVEMSAVVYKDWVFTDQALPADLIKRGMAIEDPSCPHG 598

LOX-1 601 VRLLIEDYPYAADGLEIWAAIKTWVQEYVPLYYARDDDVKNDSELQHWWKEAVEK 655
1IK3  599 IRLVIEDYPYTVDGLEIWDAIKTWVHEYVFLYYKSDDTLREDPELQACWKELVEV 653


LOX-1 656 GHGDLKDKPWWPKLQTLEDLVEVCLIIIWIASALHAAVNFGQYPYGGLIMNRPTA 710
1IK3  654 GHGDKKNEPWWPKMQTREELVEACAIIIWTASALHAAVNFGQYPYGGLILNRPTL 708
                                                 *    *
LOX-1 711 SRRLLPEKGTPEYEEMINNHEKAYLRTITSKLPTLISLSVIEILSTHASDEVYLG 765
1IK3  709 SRRFMPEKGSAEYEELRKNPQKAYLKTITPKFQTLIDLSVIEILSRHASDEVYLG 763

LOX-1 766 QRDNPHWTSDSKALQAFQKFGNKLKEIEEKLVRRNNDPSLQGNRLGPVQLPYTLL 820
1IK3  764 ERDNPNWTSDTRALEAFKRFGNKLAQIENKLSERNNDEKLR-NRCGPVQMPYTLL 817

LOX-1 821 YPSSEEGLTFRGIPNSISI                                     839
1IK3  818 LPSSKEGLTFRGIPNSIS-                                     835
```

Figure S37: Alignment of soybean LOX-3 (1IK3) and soybean LOX-1 sequences showing 73.13 % identities. Essential sites for iron binding are denoted by asterisk (*) and amino acids at the binding site are shown in red colour.

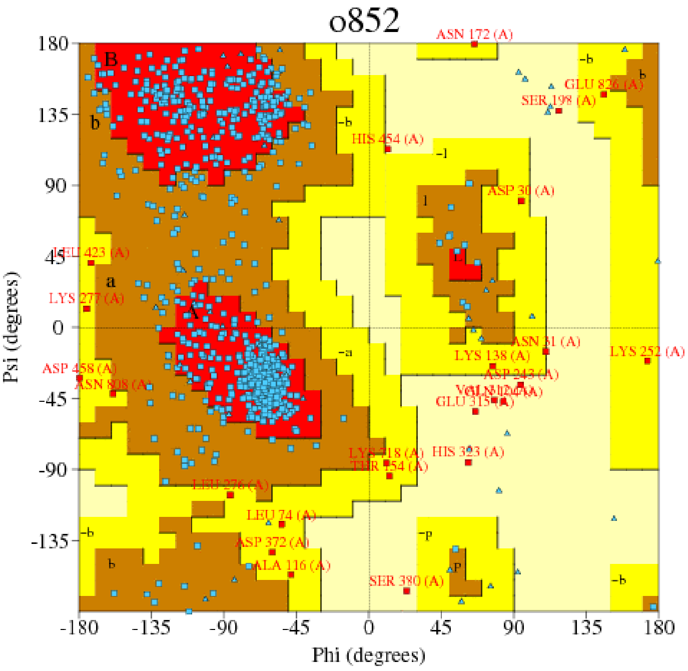

Figure S38: Ramachandran Plot of LOX-1 model produced by PROCHECK after homology modeling. [A, B,L] most favoured region; [a,b,l,p] additional allowed regions; [~a,~b,~l,~p] generously allowed regions; milky coloured areas are disallowed regions.

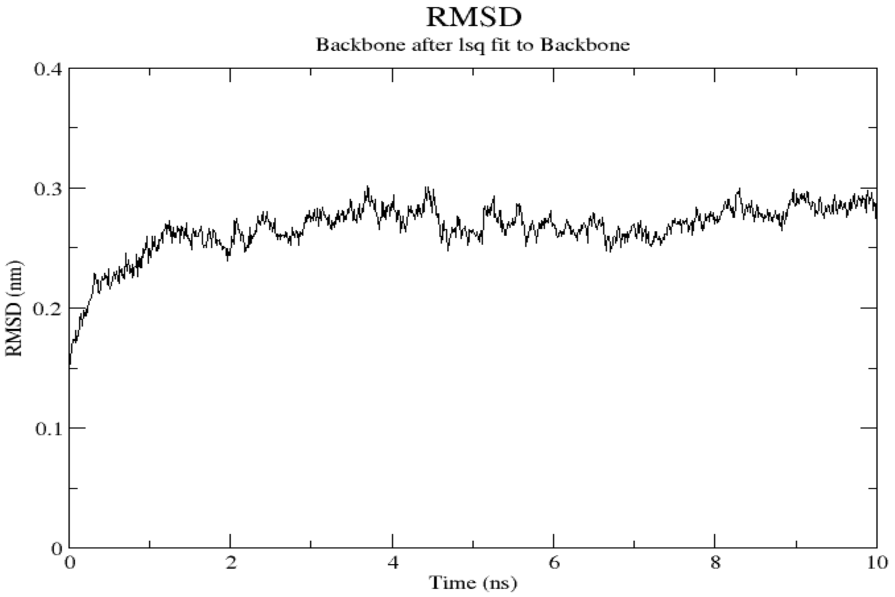

Figure S39: Total RMSD evolution along the simulation time of a protein-ligand complex of compound **3e**.

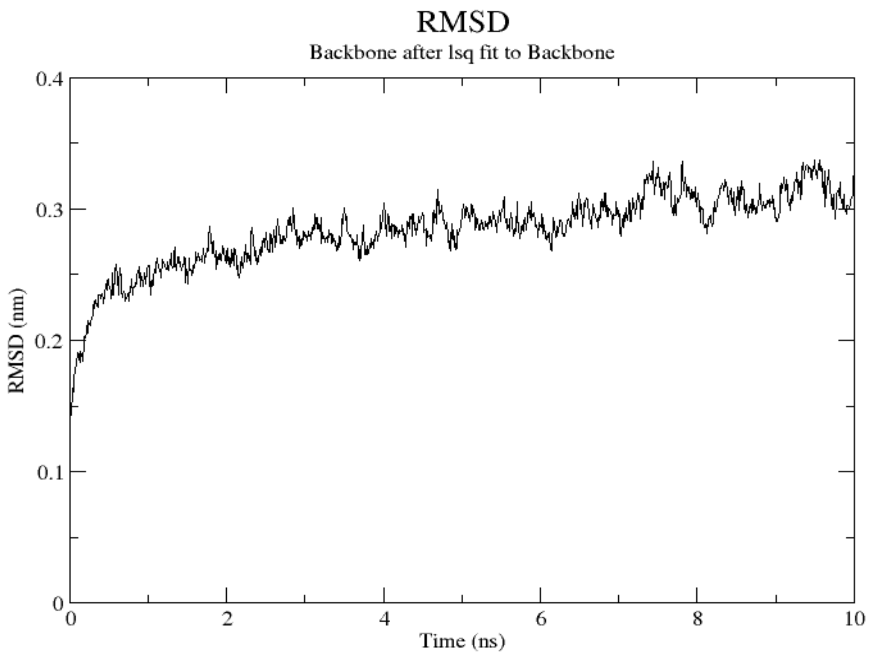

Figure S40: Total RMSD evolution along the simulation time of a protein-ligand complex of compound **4e**

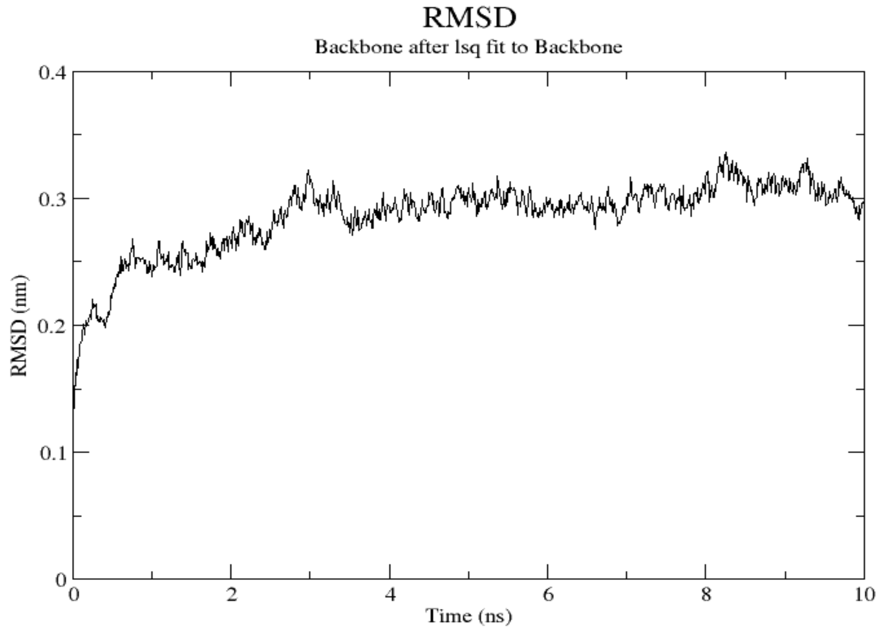

Figure S41: Total RMSD evolution along the simulation time of a protein-ligand complex of tHGA (**3a**).

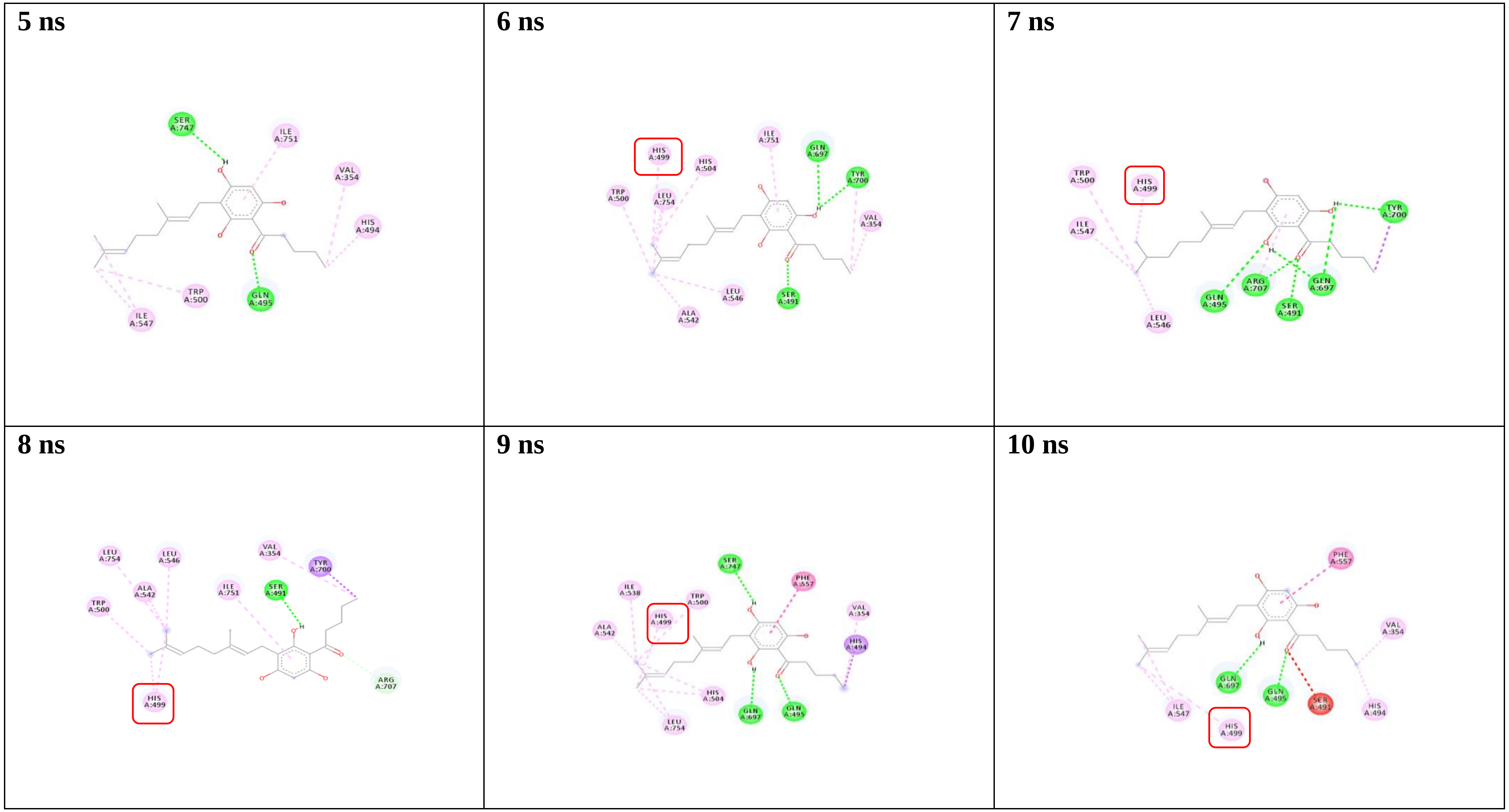

Figure S42: The 2D representation of the MD simulation result of compound 3e (snapshots taken during 5-10ns)

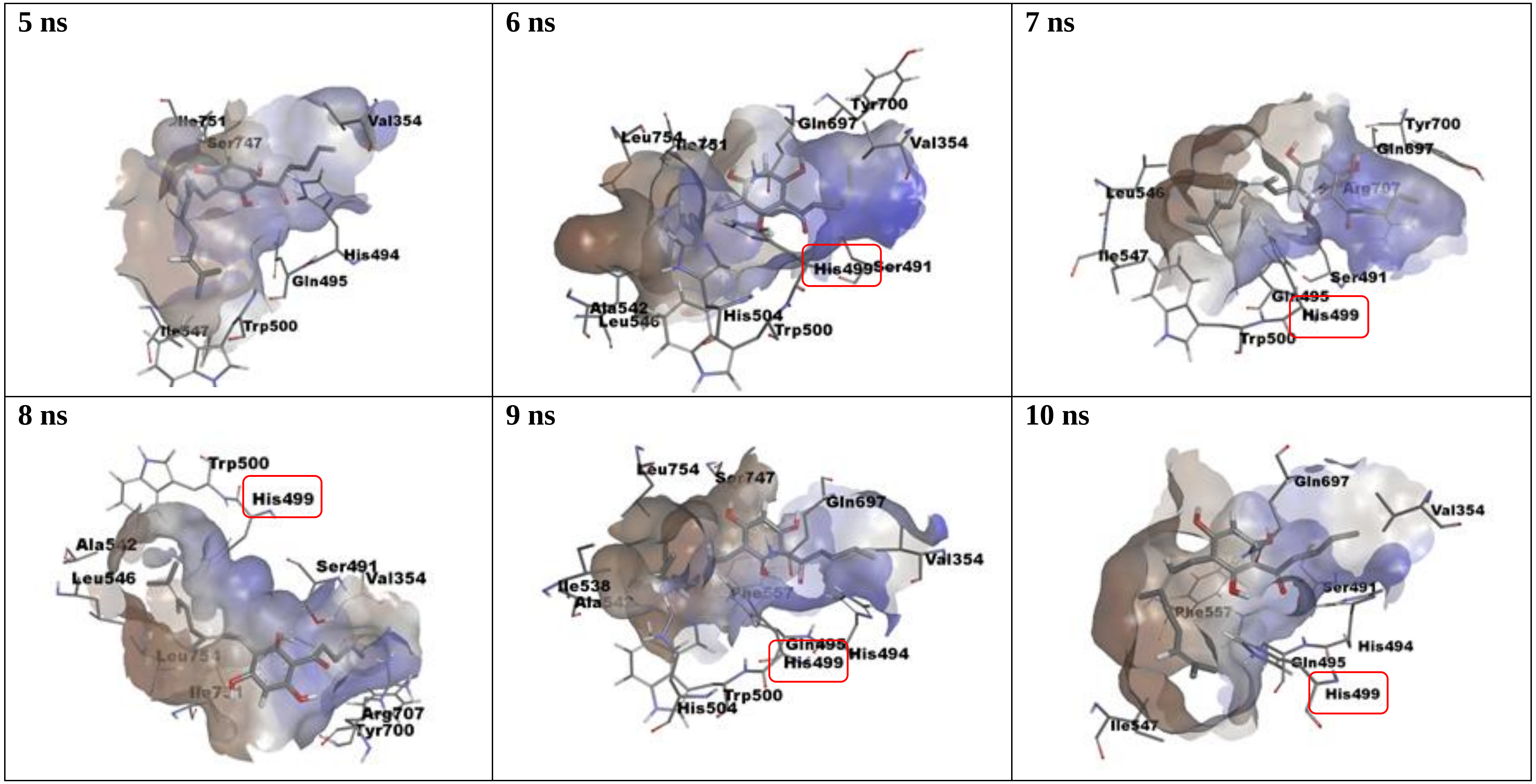

Figure S43: The 3D representation of the MD simulation result of compound 3e (snapshots taken during 5-10ns)

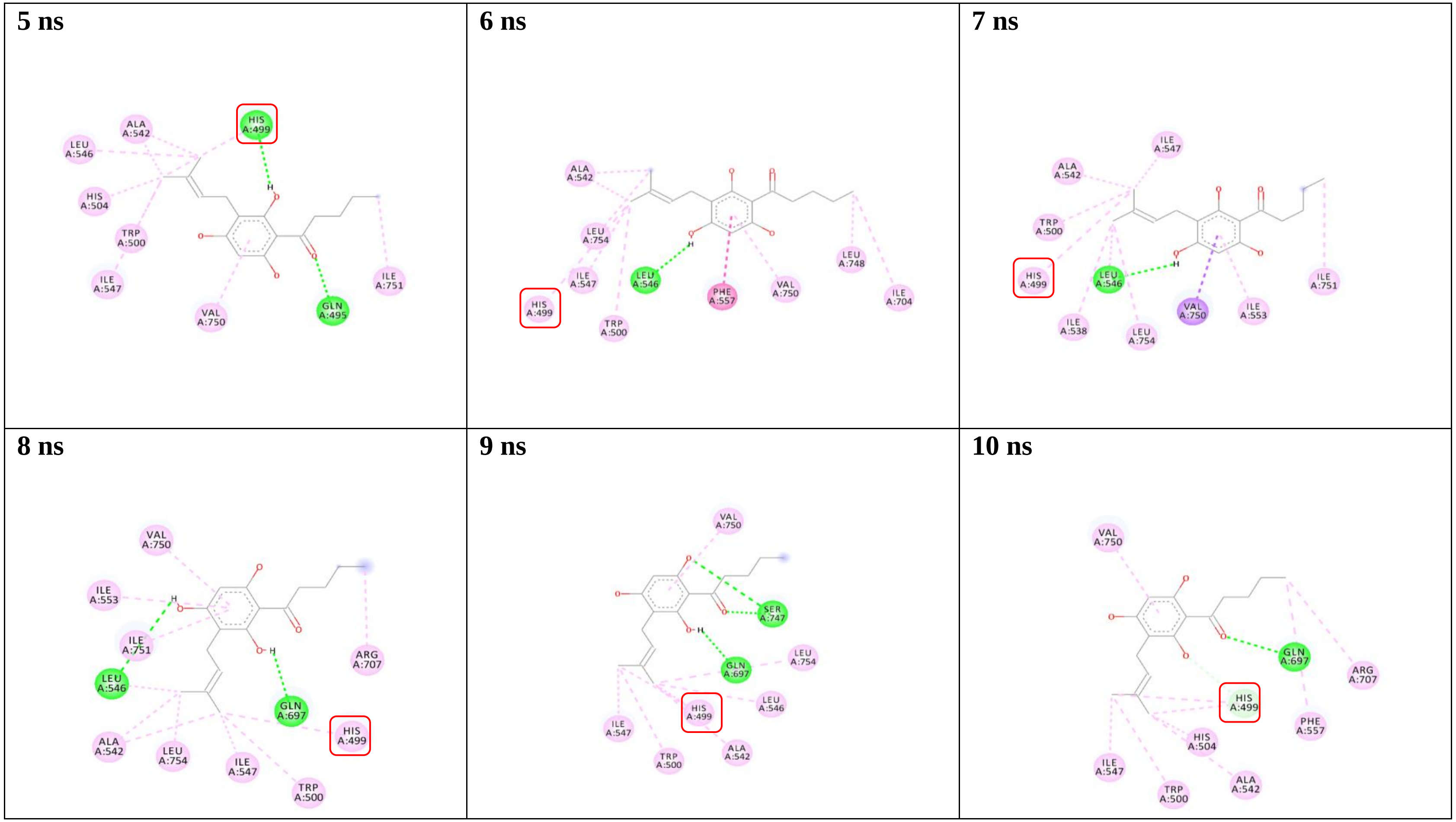

Figure S44: The 2D representation of the MD simulation result of compound 4e (snapshots taken during 5-10ns)

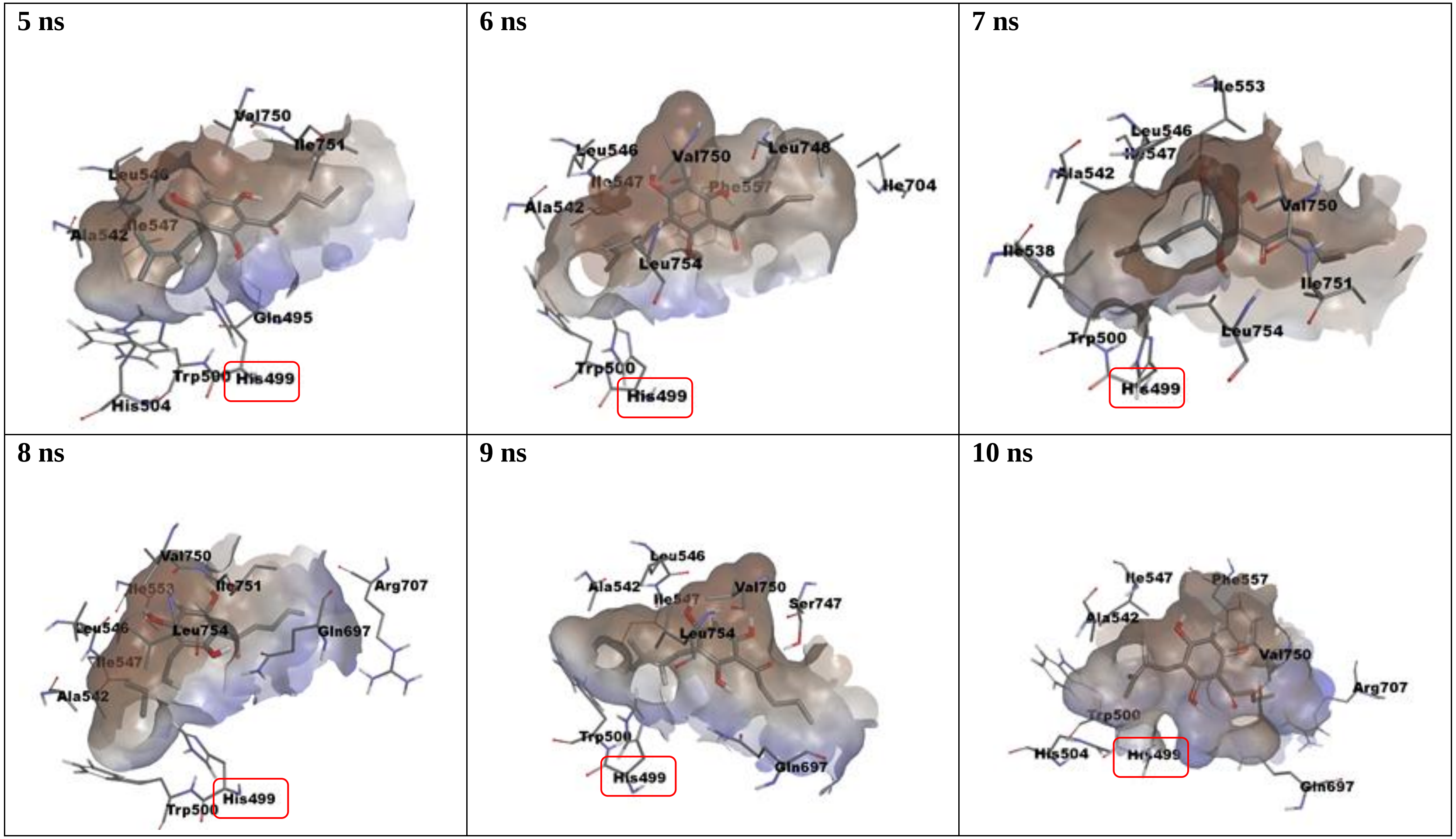

Figure S45: The 3D representation of the MD simulation result of compound 4e (snapshots taken during 5-10ns)

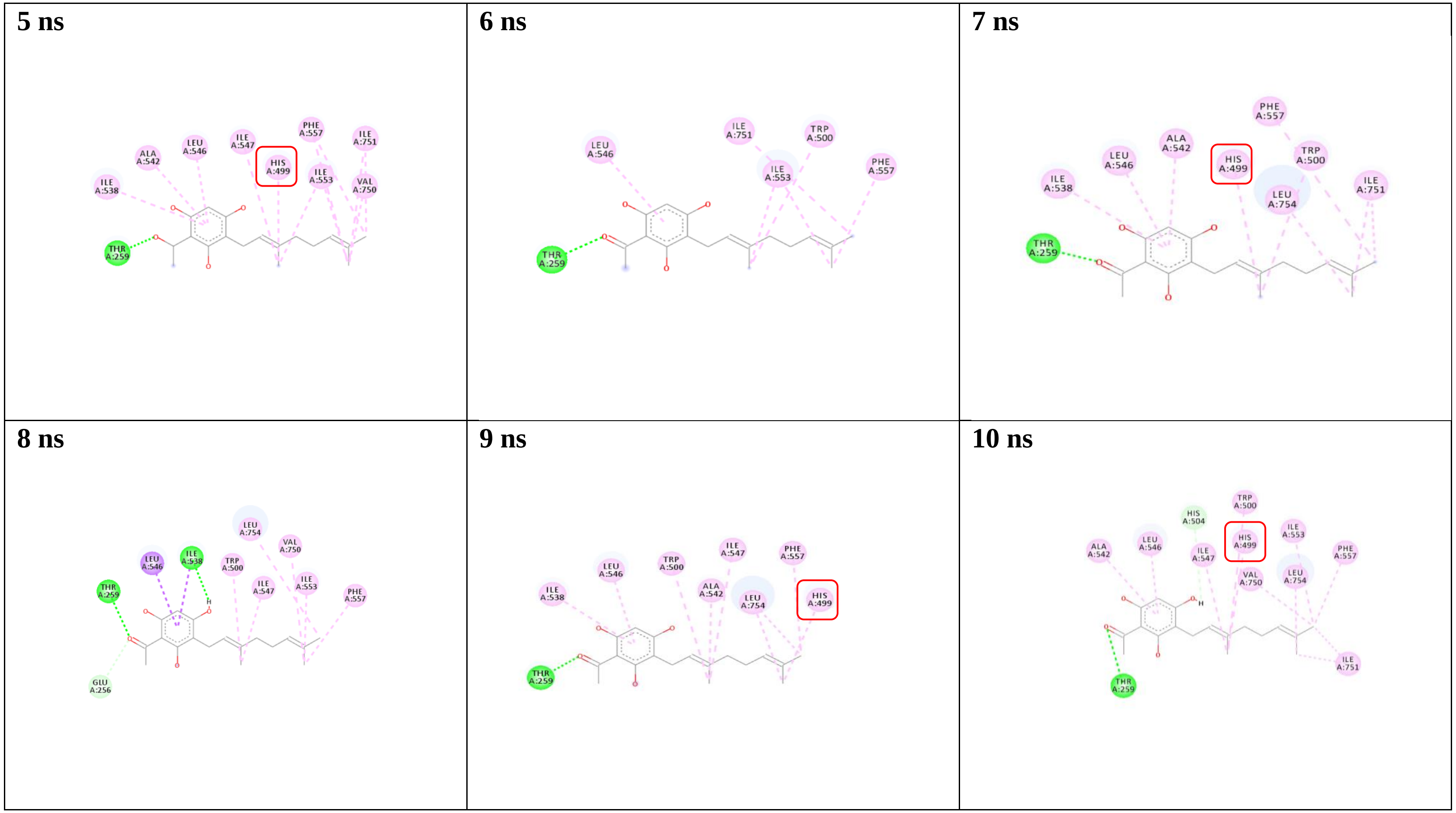

Figure S46: The 2D representation of the MD simulation result of tHGA (3a) (snapshots taken during 5-10ns)

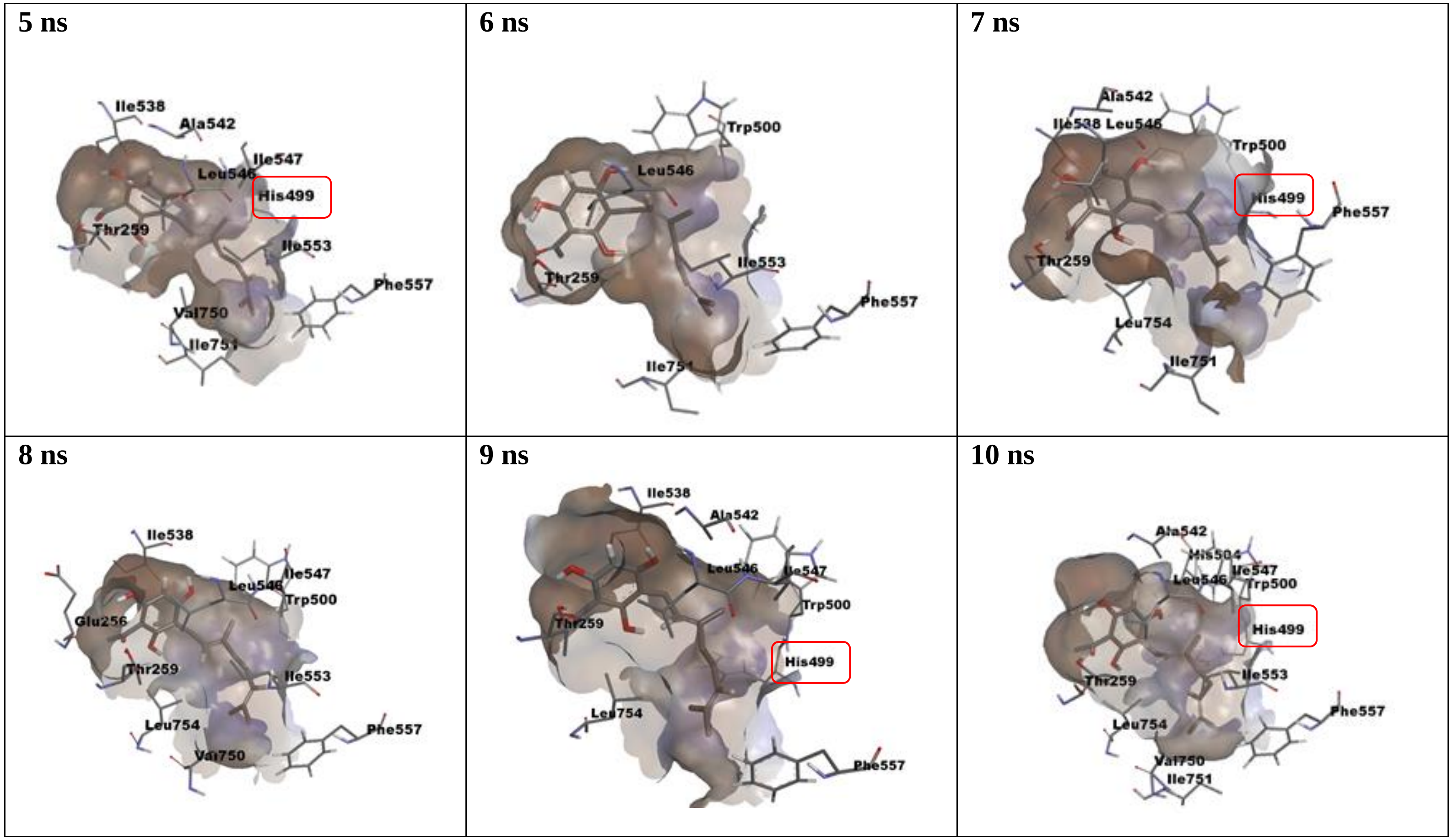

Figure S47: The 3D representation of the MD simulation result of tHGA (3a) (snapshots taken during 5-10ns)

Table 1 Residue-based decomposition of the interaction energies (kcal/mol) for molecular mechanic (ΔE_{MM}), polar solvation (ΔG_{PB}), non-polar solvation (ΔG_{SA}) and free binding energy (ΔG_{bind}) between the most active compound **3e** and the residues in the binding pocket of soybean LOX-1 model.

Residue Number	ΔE_{MM}	ΔG_{PB}	ΔG_{SA}	ΔG_{bind}
**Ser491**	-4.35 (0.12)	3.65 (0.17)	-0.51 (0.02)	-1.22 (0.15)
**His494**	-4.76 (0.19)	4.78 (0.23)	-0.41 (0.02)	-0.39 (0.24)
**Gln495**	-12.58 (0.26)	12.89 (0.39)	-0.70 (0.02)	-0.39 (0.28)
**His499**	-4.15 (0.12)	8.08 (0.33)	-0.55 (0.02)	3.39 (0.29)
**Trp500**	-2.89 (0.11)	0.84 (0.04)	-0.31 (0.01)	-2.36 (0.11)
**His504**	-1.70 (0.09)	1.79 (0.10)	-0.09 (0.01)	0.00 (0.12)
**Ile538**	-0.49 (0.05)	-0.47 (0.03)	-0.05 (0.01)	-1.03 (0.05)
**Leu541**	-0.22 (0.01)	-0.20 (0.01)	0.00 (0.00)	-0.41 (0.02)
**Leu546**	-4.96 (0.12)	1.41 (0.08)	-0.49 (0.02)	-4.04 (0.13)
**Ile547**	-2.93 (0.13)	1.01 (0.08)	-0.34 (0.02)	-2.27 (0.14)
**Ile553**	-2.05 (0.07)	0.27 (0.06)	-0.15 (0.01)	-1.94 (0.09)
**Phe557**	-8.18 (0.17)	1.94 (0.07)	-0.90 (0.02)	-7.14 (0.18)
**Gln697**	-11.77 (0.25)	14.27 (0.34)	-1.05 (0.02)	1.47 (0.31)
**Tyr700**	-2.00 (0.07)	1.36 (0.11)	-0.20 (0.01)	-0.85 (0.07)
**Arg707**	0.48 (0.08)	9.91 (0.28)	-0.29 (0.01)	10.10 (0.27)
**Ser747**	-3.52 (0.19)	4.51 (0.15)	-0.47 (0.02)	0.50 (0.19)
**Ile751**	-4.92 (0.14)	0.34 (0.07)	-0.55 (0.02)	-5.13 (0.15)
**Leu754**	-2.86 (0.11)	0.39 (0.02)	-0.37 (0.02)	-2.85 (0.12)
**Ile839**	0.26 (0.01)	-1.33 (0.02)	0.00 (0.00)	-1.07 (0.02)

Table 2 Residue-based decomposition of the interaction energies (kcal/mol) for molecular mechanic (ΔE_{MM}), polar solvation (ΔG_{PB}), non-polar solvation (ΔG_{SA}) and free binding energy (ΔG_{bind}) between compound **4e** and the residues in the binding pocket of soybean LOX-1 model.

Residue Number	ΔE_{MM}	ΔG_{PB}	ΔG_{SA}	ΔG_{bind}
**Glu495**	-9.40 (0.21)	20.18 (0.42)	-1.11 (0.02)	9.66 (0.30)
**His499**	-5.06 (0.11)	10.93 (0.27)	-0.42 (0.01)	5.45 (0.25)
**Trp500**	-3.11 (0.08)	1.43 (0.08)	-0.30 (0.01)	-1.98 (0.07)
**His504**	-1.78 (0.06)	3.50 (0.14)	-0.02 (0.05)	1.70 (0.10)
**Ile538**	-1.70 (0.06)	-0.24 (0.05)	-0.15 (0.01)	-2.09 (0.07)
**Ala542**	-1.88 (0.07)	-0.03 (0.05)	-0.21 (0.02)	-2.11 (0.09)
**Leu546**	-7.35 (0.14)	3.30 (0.10)	-0.47 (0.02)	-4.51 (0.17)
**Ile547**	-5.52 (0.12)	0.80 (0.04)	-0.46 (0.02)	-5.18 (0.11)
**Ile553**	-3.64 (0.11)	0.06 (0.02)	-0.34 (0.01)	-3.92 (0.11)
**Phe557**	-5.56 (0.13)	2.89 (0.08)	-0.63 (0.02)	-3.30 (0.15)
**Gln697**	-3.97 (0.13)	9.00 (0.27)	-0.52 (0.02)	4.51 (0.22)
**Ile704**	-0.21 (0.01)	0.01 (0.01)	0.00 (0.00)	-0.21 (0.01)
**Arg707**	-0.93 (0.08)	6.59 (0.37)	-0.30 (0.02)	5.36 (0.36)
**Ser747**	-3.20 (0.14)	3.44 (0.20)	-0.30 (0.02)	-0.07 (0.13)
**Leu748**	-0.51 (0.04)	-0.06 (0.02)	-0.01 (0.00)	-0.57 (0.03)

Table 3 Residue-based decomposition of the interaction energies (kcal/mol) for molecular Mechanic (ΔE_{MM}), polar solvation (ΔG_{PB}), non-polar solvation (ΔG_{SA}) and free binding energy (ΔG_{bind}) between tHGA (**3a**) and the residues in the binding pocket of soybean LOX-1 model.

Residue Number	ΔE_{MM}	ΔG_{PB}	ΔG_{SA}	ΔG_{bind}
**Glu256**	-1.85 (0.12)	-4.90 (0.15)	-0.14 (0.01)	-6.90 (0.12)
**Thr259**	-6.94 (0.19)	7.76 (0.11)	-0.31 (0.01)	0.51 (0.21)
**His499**	-4.21 (0.10)	7.38 (0.21)	-0.68 (0.01)	-2.49 (0.18)
**Trp500**	-3.96 (0.10)	0.92 (0.04)	-0.40 (0.01)	-3.44 (0.11)
**His504**	-4.27 (0.13)	4.38 (0.16)	-0.30 (0.01)	-0.19 (0.13)
**Ile538**	-5.97 (0.19)	-0.22 (0.07)	-0.44 (0.01)	-6.63 (0.19)
**Ala542**	-1.72 (0.16)	0.28 (0.09)	-0.26 (0.01)	-1.70 (0.16)
**Leu546**	-8.13 (0.17)	1.55 (0.05)	-0.87 (0.02)	-7.45 (0.16)
**Ile547**	-2.34 (0.08)	1.26 (0.05)	-0.33 (0.01)	-1.41 (0.09)
**Ile553**	-3.99 (0.09)	0.34 (0.02)	-0.40 (0.01)	-4.05 (0.09)
**Phe557**	-2.03 (0.07)	0.60 (0.04)	-0.14 (0.01)	-1.57 (0.07)
**Val750**	-2.09 (0.06)	-0.11 (0.03)	-0.10 (0.01)	-2.31 (0.07)
**Ile751**	-1.72 (0.06)	-0.28 (0.04)	-0.25 (0.01)	-2.25 (0.08)
**Leu754**	-8.38 (0.17)	0.90 (0.07)	-1.24 (0.02)	-8.71 (0.16)
